# Implementation challenges for achieving universal health coverage through social health protection scheme: what can we learn from Bangladesh?

**DOI:** 10.1080/17482631.2026.2623094

**Published:** 2026-02-07

**Authors:** Mohammad Wahid Ahmed, Quazi Nazmus Sakib, Md. Zahid Hasan, Gazi Golam Mehdi, Jahangir A.M. Khan, Ziaul Islam, Sayem Ahmed

**Affiliations:** a Health Economics and Financing, Health Systems and Population Studies Division, International Centre for Diarrhoeal Disease Research, Dhaka, Bangladesh; b NORDIK Institute, Sault Ste. Marie, Ontario, Canada; c Health Economics, Policy and Evaluation Research (HEPER), School of Public Health and Community Medicine, University of Gothenburg, Gothenburg, Sweden; d Health Economics and Policy Research Group, Department of Learning, Informatics, Management and Ethics, Karolinska Institute, Solna, Sweden; e Department of International Public Health, Liverpool School of Tropical Medicine, Liverpool, United Kingdom; f Department of Health Sciences, Brunel University London, Uxbridge, London, UK

**Keywords:** Social health protection scheme, implementation challenges, below-poverty-line population, health financing, universal health coverage, Bangladesh

## Abstract

**Background:**

In Bangladesh, households experience high out-of-pocket healthcare expenditure, with below-poverty-line population being disproportionately affected. To reduce financial hardship, the government piloted a social health protection scheme targeting poor households in selected sub-districts. This study examined the implementation barriers of the scheme.

**Method:**

A mixed-methods design was applied. Quantitative data were collected through survey of enrolled households (*n* = 806). The qualitative component comprised KIIs (*n* = 10) with scheme implementers and healthcare providers, and FGDs (*n* = 5) with beneficiaries.

**Results:**

Household survey indicated low service utilization (16.1%) among cardholders. Awareness of specific benefits was also limited, with only 19.1 percent aware of free diagnostics and 9.4 percent aware of free referrals. Qualitative findings confirmed these demand-side barriers, highlighting inadequate knowledge of beneficiaries, dissatisfaction with care quality, and negligence in service delivery. Key supply-side challenges included staff shortages, low provider motivation, and delays in claim settlement. The absence of outpatient coverage emerged as a common concern across stakeholders. At the ecosystem level, weak local-level coordination and rigid public financial rules further hindered implementation.

**Conclusion:**

Implementation challenges were largely systemic, reflecting misalignment between program design and operational realities. Addressing these challenges is essential to ensure the success of future initiatives in Bangladesh and comparable settings.

## Introduction

Out-of-pocket (OOP) health expenditures in Bangladesh have soared to 72.5% of total health spending, a direct consequence of the combined effect of a rising burden of non-communicable diseases, escalating healthcare costs, and the absence of a proper financing mechanism (Hassan et al., 2016; Rawal et al., 2019; The World Bank, 2022). This high level of OOP spending has a catastrophic economic impact on households, particularly those with low incomes, pushing many into poverty (National Academies of Sciences, Engineering, and Medicine, 2018; United Nations General Assembly, 2015). At the same time, the Government of Bangladesh (GoB) affirmed health as a fundamental human right and is committed to achieving universal health coverage (UHC), as outlined in the National Health Policy 2011, by ensuring access to affordable health services for all (Ministry of Health and Family Welfare, 2011).

To achieve these goals, the government adopted the Health Care Financing Strategy 2012–2032, which aims to provide all citizens with financial protection for healthcare by 2032 (Health Economics Unit, 2012). A key initiative under this strategy was the development of the *Shasthyo Suroksha Karmasuchi* (SSK), a social health protection scheme, implemented by the Health Economics Unit (HEU) of the Ministry of Health and Family Welfare (MoHFW) with the support from German Development Cooperation through KfW (German Development Bank) and GFA Consulting Group. Adopting the mechanism of health insurance model, the scheme was developed over a three-year period of extensive consultations with the experts. As a pilot project, the scheme was first implemented in one upazila (sub-district) of Tangail district. The enroled members were entitled to inpatient healthcare services at the designated Upazila Health Complex (UpHC) and structured referral care at the district hospital. Each participating household received an electronic health card that provided healthcare coverage worth 50,000 BDT per year for 70 specified disease groups. The annual premium of 1,000 BDT for this coverage was fully subsidised by the government. Compared with regular patients at public health facilities, SSK members benefited from several advantages, including free outpatient consultations, free inpatient and referral care, free ambulance service from designated UpHC to district hospital, access to grievance redress mechanisms for service quality issues, and free essential medicines at both UpHC and district hospital level for inpatient treatment. The HEU designed and oversaw the scheme, while the SSK Cell managed its implementation, policy coordination, and monitoring. The Scheme Operator (SO) handled operational activities, including distributing health cards to below-poverty-line (BPL) households and supporting healthcare delivery and claim processing at the designated hospital (Ahmed et al., 2018). During the pilot phase, only BPL households, identified through community-based poverty targeting, were included. The inclusion of above-poverty-line (APL) households was planned for later phases to enhance programme sustainability (SSK Cell, Health Economics Unit, Minsitry of Health & Family Welfare, 2014). Although the initiative was subsequently expanded to several upazilas across Bangladesh, it remained a pilot programme until its suspension in 2024 (Hamid, 2024).

Since its launch, several studies have examined the SSK scheme from various perspectives. The existing literature has mainly evaluated patient satisfaction with healthcare services, identified factors affecting service utilisation, measured effects on financial hardship, and the importance of the nationwide scaling up of the scheme (Hasan et al., 2022, 2024a; Khatun et al., 2025). The scheme was found effective in reducing financial risks for the BPL population, significantly lowering OOP expenditures, catastrophic health expenditure, and impoverishment (Hasan et al., 2024a, 2024b). Despite this, overall utilisation of the scheme was low, with studies indicating that as few as 8% of enroled members who sought care did so through SSK (Hasan et al., 2022). Key barriers to service utilisation included lack of knowledge about the scheme's benefits, limitations in the BPL household identification process, long within-facility waiting times for care and poor facility amenities (Hasan et al., 2022, 2024b). While these studies provided valuable insights into specific facets of the scheme, a comprehensive study that holistically investigates the full spectrum of demand-side, supply-side and ecosystem-related implementation challenges of SSK from the perspectives of all key actors has been lacking. An investigation into the demand-side challenges focusing on barriers to accessing and receiving proper services, and the supply-side challenges addressing constraints to efficient service delivery, would help reveal how these limitations collectively shaped the scheme's performance. The ecosystem challenges, on the other hand, would illustrate how administrative, managerial, and planning-related barriers across key institutional actors, including the HEU, the SSK Cell, and the SO, constrained coordination, accountability, and effective implementation of the scheme.

A comprehensive study addressing the implementation challenges of SSK is particularly important as all the operations of this programme were suspended effective July 1, 2024. This suspension is a result of the expiration of the 4th Health, Population, and Nutrition Sector Programme (4th HPNSP) and a delay in the timely initiation of the subsequent fifth sector programme (Hamid, 2024). A thorough analysis of the implementation challenges is therefore essential, both to guide the refinement of future initiatives in Bangladesh and to serve as a valuable case study for similar initiatives in other low-resource settings. To fulfil this objective, this study adopts a qualitatively-driven mixed-methods design to explore and understand the demand-side, supply-side and ecosystem barriers affecting the implementation of the SSK scheme in Bangladesh.

## Materials and methods

The previously published protocol for this study provides a detailed description of the SSK scheme, including its management body, operator, benefit package, claim management process, and information management system. The study setting, sampling strategy for both qualitative and quantitative surveys, and data collection instruments are also detailed in the protocol (Ahmed et al., 2018). Hence, the key methodological components relevant to this particular study are described in this section.

### Study setting

The study was conducted in an upazila located in a district in central Bangladesh, which served as one of the initial pilot sites for the SSK scheme. As of 2019, the total number of households in the selected upazila was approximately 89,351, of which 35,740 BPL households were enroled in the SSK scheme (Ahmed et al., 2018). This upazila was selected for the study because, at the time of data collection in 2018, the scheme was comparatively more mature there than in other pilot sites.

### Study design

A mixed-methods sequential explanatory design was employed to examine the implementation-related challenges of the SSK scheme. The sequential explanatory design involves collecting and analysing quantitative data, followed by qualitative enquiry to expand upon the initial quantitative results (Poth, 2023). The quantitative component comprised a community survey administered to the enroled households of the scheme to assess challenges related to beneficiaries' knowledge, awareness, and utilisation. The qualitative component, which involved key informant interviews (KIIs) and focus group discussions (FGDs), provided an in-depth understanding of the supply-side and ecosystem barriers as well as detailed the demand-side challenges, respectively. This particular study design was selected since it allowed to quantify the extent of awareness and utilisation gaps, while the qualitative part explained the underlying reasons and contextual implementation challenges. This approach was considered methodologically appropriate for assessing the implementation and contextual dynamics of a community-based health intervention like the SSK scheme.

### Community survey

A cross-sectional community survey was administered to assess cardholders' knowledge and awareness of the SSK scheme and also to document barriers to service utilisation. A two-phase sampling strategy was employed to select the target households. First, the 15 unions of the selected upazila were stratified based on their proximity (near, medium, or far) to the local health complex. Given the unequal distribution of enroled households across these unions, the sample was allocated using a probability proportional to size method. In the second phase, specific households within each union were selected through simple random sampling.

Data were collected between July and September 2018 using a standard, pre-tested questionnaire, which was administered by a trained six-member research team after obtaining informed written consent. Household heads were targeted as the primary respondents. If the household head was temporarily unavailable, enumerators scheduled a revisit at a convenient time. When the household head was not available at all, another household member aged 18 years or older was interviewed. The survey instrument collected information on households' socio-demographic characteristics, illness and treatment history, and knowledge of the SSK benefit package.

The required sample size for the community survey was determined based on the expected healthcare utilisation rate from a comparable health protection scheme for a BPL population. The calculation used a 19% utilisation rate reported for India's *Rashtriya Swasthya Bima Yojana* (RSBY) scheme (Philip et al., 2016). Since the total enroled population for the scheme was 35,740, this yielded an initial requirement of 657 households, based on a 95% confidence interval and a 3% margin of error. The target sample was subsequently adjusted to 828 households to account for an anticipated 5% non-response rate and a 1.2 design effect for cluster sampling. Due to time constraints during fieldwork, a final sample of 806 households was successfully interviewed in the selected upazila.

### Key informant interviews (KIIs)

Ten KIIs were conducted with stakeholders that included SSK service providers, scheme management personnel, and personnel from the Health Economics Unit in order to investigate the systemic challenges that impeded the implementation of the SSK project and to solicit expert recommendations. We developed distinct interview guidelines for personnel from three key groups, namely, service providers, insurance personnels, and personnels from HEU, to reflect their unique roles within the scheme (see Supplementary Material).

### Focus group discussions (FGD)

The study employed five FGDs to gain an in-depth understanding of beneficiary perspectives on the SSK scheme. The primary objectives were to assess their knowledge of available services, utilisation experiences, the perceived quality of care, and barriers to accessing care. A two-phase sampling strategy was utilised for recruitment. Initially, the 15 unions of the selected upazila were geographically stratified based on distance to the local health complex. Subsequently, participants were purposively selected from these strata. 8 to 10 individuals participated in each FGD. Both male and female individuals aged 18 years and above were eligible to participate. To ensure diversity of perspectives, each discussion group included participants who had used the SSK card and those who were enroled but had not utilised it. This sampling strategy was intended to capture variations in awareness, utilisation behaviour, and perceived challenges between different beneficiary sub-groups.

Each FGD was conducted following a standardised discussion guideline (see Supplementary Material). A trained facilitator moderated the sessions, ensuring that all participants had opportunities to contribute sequentially while also encouraging interaction and discussion among them. The facilitator used open-ended questions and probing techniques to stimulate group dialogue, clarify responses, and validate emerging viewpoints. Although certain participants contributed more actively due to differences in knowledge or experience, the moderator encouraged cross-participant reflections to elicit agreement, disagreement, or complementary perspectives.

### Analysis method

Descriptive analysis was performed using quantitative data. In this analysis, the level of knowledge, awareness, and service utilisation was explored. Regarding qualitative analysis, a verbatim transcription and translation were performed immediately after completion of a KII and FGD using the audiotapes and interview notes. A systematic framework approach was employed to analyse the qualitative data from KIIs and FGDs and to generate appropriate themes and codes. The framework method supports thematic analysis in a systematic manner for organisation and mapping the qualitative interview data, which is appropriate for interdisciplinary and collaborative scheme projects (Gale et al., 2013). Two research team members (MWA, QNS) familiarised themselves with the KII and FGD data by repeatedly reading the transcripts. Following this familiarisation, they systematically coded the transcripts to identify emergent issue areas and themes. For the analysis, a framework matrix was generated using a spreadsheet, into which the data were summarised and charted by issue area and theme. This charting process ensured that data were condensed while preserving the participants' original expressions and opinions prior to interpretation. Direct quotes were documented under their corresponding themes where deemed appropriate. Finally, the interpreted findings under each issue area were presented to identify key implementation barriers.

### Ethical approval

Informed written consent was obtained from all participants prior to enrolment, and confidentiality and anonymity of their data were strictly maintained. Ethical approval for the study was granted by the Research Review Committee and the Ethics Review Committee of the International Centre for Diarrhoeal Disease Research, Bangladesh (icddr,b) (Protocol No. PR-17047).

## Results

### Quantitative findings

Table I presents the findings from the community survey regarding beneficiaries' knowledge, awareness, and utilisation of the SSK scheme. Among the surveyed households (*n* = 806), a vast majority reported having experienced catastrophic health expenditure (97.3%) and observed neighbours working despite illness (98.3%), while only 27.5% were aware of other institutions that help bear treatment costs. General awareness of health insurance was low (13.8%); however, a majority of households (67.7%) had heard of the SSK scheme.

**Table I.** Beneficiary's knowledge, awareness, and utilisation of the SSK scheme.

Characteristic	Response, *n* (%)		Confident Interval (95%)
**Healthcare context and perceptions**			
**Experienced Catastrophic Health Expenditure (CHE)**			
Yes	784 (97.3)	(95.8–98.2)	
No	22 (2.7)	(1.8–4.1)	
**Neighbours used to work despite being sick**			
Yes	792 (98.3)	(97.1–98.9)	
No	14 (1.7)	(1.0–2.9)	
**Different institutions used to bear treatment cost of their members**			
Yes	222 (27.5)	(24.6–30.7)	
No	584 (72.5)	(69.3–75.4)	
**Awareness and knowledge of health insurance and SSK**			
**Ever heard of health insurance**			
Yes	111 (13.8)	(11.6–16.3)	
No	695 (86.2)	(83.7–88.4)	
**Heard about the local SSK protection scheme for the poor people**			
Yes	546 (67.7)	(64.4–70.9)	
No	260 (32.3)	(29.1–35.6)	
**Perceived mode of SSK service access**			
Through card	508 (63.0)	(59.6–66.3)	
In exchange of cash	1 (0.1)	(0.01–0.88)	
Don't know	297 (36.8)	(33.6–40.2)	
**Knowledge of free outpatient registration service**			
Yes	361 (44.8)	(41.4–48.3)	
No	8 (0.9)	(0.5–1.9)	
Don't know	437 (54.2)	(50.8–57.6)	
**Knowledge of free medicine**			
Yes	358 (44.4)	(41.0–47.8)	
No	51 (6.3)	(4.8–8.2)	
Don't know	397 (49.2)	(45.8–52.7)	
**Knowledge of free diagnostic services**			
Yes	154 (19.1)	(16.5–21.9)	
No	47 (5.8)	(4.4–7.7)	
Don't know	701 (75.1)	(71.9–77.9)	
**Knowledge of free referral facility to district hospital**			
Yes	71 (9.4)	(7.6–11.7)	
No	29 (3.6)	(2.5–5.1)	
Don't know	705 (86.9)	(84.5–89.1)	
**Knowledge of free transportation cost to referral hospital**			
Yes	71 (8.8)	(7.0–10.9)	
No	30 (3.7)	(2.6–5.3)	
Don't know	705 (87.5)	(84.9–89.6)	
**Sources of information on the SSK scheme**			
From others in the locality	232 (41.7)	(37.7–45.9)	
Representative from SSK	527 (94.8)	(92.6–96.4)	
From familiar person	160 (28.8)	(25.2–32.7)	
From local leader	16 (2.9)	(1.7–4.7)	
From miking	1 (0.2)	(0.03–1.27)	
Others	7 (1.3)	(0.6–2.62)	
**Utilisation of SSK services**			
**Ever used the SSK card**			
Yes	130 (16.1)	(13.7–18.8)	
No	676 (83.9)	(81.2–86.3)	
**Reasons for not using the SSK card**			
Don't know how to use	368 (54.4)	(50.4–58.3)	
Long distance of the facility	42 (6.2)	(4.5–8.4)	
Poor transportation system	12 (1.8)	(1.0–3.2)	
Didn't have need	197 (29.1)	(25.6–32.8)	
Others	57 (8.5)	(6.5–10.9)	
**Frequency of SSK service utilisation among beneficiaries**			
One time	95 (72.3)	(63.8–79.4)	
Two times	18 (13.8)	(8.9–21.0)	
Three times	5 (3.8)	(1.5–9.0)	
Four times	5 (3.8)	(1.6–9.0)	
Five times	1 (0.8)	(0.1–5.4)	
Six times	3 (2.3)	(0.7–7.0)	
Seven times	2 (1.5)	(0.4–6.1)	
Ten times	1 (0.8)	(0.1–5.4)	

Specific knowledge of the scheme's benefits varied: 63.0% knew services were provided via a card, but less than half were aware of free outpatient services (44.8%) or free medicine (44.4%). Awareness was substantially lower for free diagnostic services (19.1%), free referral facilities (9.4%), and free transportation for referrals (8.8%). The primary source of information was a representative from SSK (94.8%), followed by others in the locality (41.7%).

Utilisation of the scheme was low, with 83.9% of respondents reporting they had never used their card. The most common reason for non-use was not knowing how to use the card (54.4%), followed by not having a need for the service (29.1%). Among the minority of beneficiaries (*n* = 130) who did utilise the scheme, most (72.3%) did so only a single time.

### Qualitative findings

Our findings from qualitative aspects focused on the implementation related challenges of SSK. These challenges were focused on the demand side (SSK beneficiaries' aspect), the supply side (service provider aspects) and the ecosystem (administrative and managerial aspects).

### Demand-side challenges

Thematic analysis of the FGDs transcripts identified five broad demand-side issue areas, which collectively encompass 14 distinct themes, as summarised in Table II.

**Table II.** Demand-side challenges identified through FGDs.

Issue areas	Identified themes	Summary results
Poor knowledge of cardholders	Poor overall knowledge about SSK scheme	Unaware of inpatient-only focus and free referral benefits, due to inadequate promotion.
	Poor knowledge of the benefit package	Lacked clarity on coverage for specific diseases, medicines, and diagnostic tests.
Erroneous registration process	No prior announcement before registration	Registration occurred without prior announcement, leading to exclusion of eligible individuals.
	Incomplete registration coverage	Not all eligible households were registered; some who registered never received a card.
Dissatisfaction with SSK services	Denied the benefits of the card	Cardholders were refused care for both covered and uncovered conditions; They were sometimes forced to pay out-of-pocket for services.
	Discrimination in service delivery	Perception that quality of care depended on personal connections with hospital staff.
	Improper conduct of service providers	Reports on staff negligence, verbal abuse, demeaning language, and unprofessional behaviour.
	Negligence in service delivery	Delayed emergency referrals and dismissal of patient concerns without diagnostic tests.
	Prolonged waiting time	Delays in receiving medication after admission and during the discharge process.
	Inappropriate facility environment	Poor sanitation, particularly extremely dirty toilet facilities.
	Ineffective and improper treatment	Treatment was often perceived as ineffective, leading to worsening conditions post-discharge.
Low motivation in seeking care	Due to refusal of care	Negative word-of-mouth from others who were refused care discouraged community members.
	Due to ineffective medicine	Perceived ineffectiveness of provided medicines and substitution of prescribed drugs by the pharmacy.
	Distance of SSK facility from BPL households	Travel costs to the facility were a significant financial deterrent, especially for minor illnesses.
Unavailability of outpatient care		Strong demand for free outpatient services; lack of it led to incomplete treatments or unnecessary hospital admissions.

1. *Poor knowledge of cardholders*

1.1. *Poor overall knowledge about SSK scheme*: Some cardholders were unaware that their eligibility for free treatment was limited to inpatient care and that they could be referred to tertiary hospitals for advanced inpatient care at no cost. Additionally, some respondents believed that the card entitled them to all types of free medication.

The FGD participants attributed the primary reasons for this limited awareness to inadequate communication from community leaders and local authorities regarding the SSK scheme, misinformation or lack of knowledge among community health workers, and insufficient promotional activities.

A beneficiary described the limited information provided during the card distribution process, stating:

*“They only talked about [free] treatment. They didn't mention anything else. No, they didn't say anything else. They just said if you take this card to that place [dedicated facility], the treatment will be free. That's all they said.”*

1.2. *Poor knowledge of the benefit package*: Cardholders had incomplete knowledge of the benefits provided by the card. While they were aware that it could be used for free treatment, many were uncertain about whether it covered medicines and diagnostic tests. Importantly, they lacked clarity on the specific diseases included in the scheme. Some assumed that the card provided free treatment for all diseases, while others had a general understanding that it covered 70 specific conditions.

One participant, recalling what they believed the card offered, stated:

*“It's written there that they will provide complete treatment for whatever illness you have.”*

A few respondents also incorrectly assumed that they would receive a partial refund for treatments of diseases not included in the list.

2. *Erroneous registration process*

2.1. *No prior announcement before registration*: Cardholders reported that the registration process in their village began abruptly, and as a result, some eligible individuals were missed because they were absent from home on that day.

One participant, explaining why many in the community did not know about the registration, stated:

*"They just came suddenly. we didn't even know when the people for the card came, which houses they went to. they came with a list."*

2.2. **I*ncomplete registration coverage*: In some areas, cardholders claimed that certain eligible families were not included in the registration process. One participant stated:

*"There are many poor people in our village who didn't go to. in our village, there were many poor people who didn't get this card."*

Additionally, some individuals reported that despite their names and information being collected, they did not receive the card. A participant described completing the registration process but never receiving the promised card:

*"We were supposed to get it. We had our pictures taken, but we didn't get the card. They took our pictures and everything, but the card that was supposed to come later never came."*

It was reported that the officials responsible for registration did not visit homes individually; instead, they gathered people in one location and provided general information. Highlighting the issues in the registration process, a respondent explained the consequences of officials not visiting homes individually:

*"If they had gone from house to house to make the list, then many people in this village would have gotten the card. They didn't come to the houses to make a proper list. they didn't do it for everyone."*

3. *Dissatisfaction with SSK services*

3.1. *Denied the benefits of the card*: Some cardholders expressed dissatisfaction after being denied treatment at the designated UpHC, despite holding an SSK card and seeking care for conditions that were covered under the scheme. In addition, several cardholders reported being refused care for fever and other conditions, which were not covered under the benefit package.

One respondent specifically mentioned being denied care for kidney problems, despite the condition being included in the benefit package. One cardholder reported that after being referred to a district hospital from the designated UpHC for an operation, he arrived with the referral slip, but the service providers at the district hospital refused to treat him.

There were also reports where cardholders had to pay out-of-pocket for treatments, as they were told the card was useless or questioned about its validity. One respondent shared his experience of paying for a blood test at the designated UpHC, stating:

*“I was suffering from a fever for many days. Then I went to the designated UpHC, showed my card, and provided a blood sample for testing. I had to pay.”*

In another instance, a cardholder required an ultrasound as part of her treatment but had to pay for it herself. Another cardholder reported having to pay for inpatient care when they visited the designated UpHC to admit their child. One respondent recalled their aunt's experience, saying:

*“My aunt-in-law went for treatment and didn't receive free care, so we decided not to seek treatment there.”*

3.2. *Discrimination in service delivery*: In some cases, respondents reported that knowing or being acquainted with officials at the facility could help secure better treatment, while those without such connections often received inadequate care. There were instances where hospital staff initially refused to provide free treatment to cardholders, however, when the cardholders approached doctors directly and made a request, they were allowed to receive free care.

A participant directly admitted their ability to receive free treatment was entirely dependent on having a personal connection at the facility:

*“I received treatment twice. I got treatment because I have a person I know there.”*

3.3. *Improper conduct of service providers:* Cardholders reported that the patients were not promptly attended to by nurses at the facility, despite multiple requests. Additionally, nurses did not adequately explain the treatment process and, at times, spoke to them in a demeaning manner, with some nurses even stating that the card was useless. Hospital staff were also reported to use foul language toward cardholders on occasion, and some respondents described the behaviour of medical technologists and clinic assistants as rude and unprofessional.

A respondent shared their experience, stating:

*“When I went to the designated UpHC, they threw my card away and told me to take it back to the people who gave it to me. They said that person would provide the medicine.”*

3.4. *Negligence in service delivery:* Multiple cardholders reported instances of negligence in care. One cardholder seeking delivery care for a critically pregnant patient described the situation and its impact, stating:

*“When we took a very critical pregnant patient for delivery at the designated UpHC, the hospital authority said they would perform a caesarean section at night. However, at 10 PM, they informed us that the patient needed to be transferred to Tangail. The hospital authority said they could manage the ambulance but requested us to arrange a driver. We had to find the driver and convince him to take the patient to Tangail.”*

Another respondent reported an instance during a caesarean section where, despite the new mother experiencing severe pain, painkillers were not administered. After multiple requests to the attending nurses, the situation was only addressed when a doctor intervened and ordered the nurses to provide painkillers.

Respondents also reported that when they sought care, they were told they were in good health and did not require treatment, despite no diagnostic tests being conducted.

One respondent said,

*“Without doing diagnostic tests, they will say you are in good health. Go home"*

3.5. *Prolonged waiting time*: Respondents reported waiting for approximately 30 minutes to receive treatment, after admission. They also experienced significant delays in receiving the medications prescribed, after being discharged, for the following seven days.

One respondent explained that the delay related to receiving medication is specifically related to sourcing the medicine from an external pharmacy:

*“…… the medicine is given a little late. They bring it from the shop…… that's what I'm saying, it takes a little while for the medicine to arrive.”*

This extended waiting time was consistently mentioned by respondents, who noted that it added to their pain and suffering.

3.6. *Inappropriate facility environment*: While participants generally described the environment of the designated UPHC as clean, they specifically complained about its toilet facilities being extremely dirty and often avoided by patients. They reported facing significant difficulty when trying to use the toilets.

One respondent stated:

*"The toilet is a bit of a problem, the toilet is not clean, you can't go in there. using the toilet for urination is a big problem."*

3.7. *Ineffective and improper treatment*: In some cases, cardholders reported receiving inpatient care, but the treatment did not cure their illness.

One respondent said,

*“I received inpatient care for one week, but the treatment didn’t cure my disease. Then I returned home, and they gave me medicine for one additional week to take at home. After returning from the hospital, my health condition deteriorated. Then I went to a different doctor [in another facility] and now I am a little better.”*

One cardholder mentioned bringing her husband for treatment, but the patient was refused admission and only provided two “Histacin” tablets.

One respondent shared their experience, stating:

*“After being admitted for 3 days, they told me to go back home even after my condition didn't improve. Then I stayed for three more days, but my condition remained the same. Then they released me with 7 days of medication. After returning home, my condition deteriorated. I had to seek care from elsewhere to recover.”*

4. *Low motivation in seeking care*

4.1. *Due to refusal of care*: Respondents reported that when their acquaintances or neighbours shared poor treatment experiences at the facility, it discouraged them and led others in the community to avoid seeking care there. One respondent mentioned that the facility staff engaged in misconduct and refused to provide treatment to cardholders, leading them to seek care elsewhere despite having diabetes and high blood pressure, both of which were included in the benefit package.

One respondent shared,

*“I avoided seeking care there after observing others who did not receive adequate treatment.”*

These incidents influenced some cardholders to seek care from elsewhere instead of visiting the designated SSK health centre.

One respondent stated,

*“If we get sick, we buy medicine from the pharmacy, but we do not visit the UpHC for treatment.”*

4.2. *Due to ineffective medicine*: Some respondents expressed concern that the designated pharmacy for cardholders didn’t dispense the prescribed medications, but instead substituted them with alternative drugs, reportedly under the influence of medical representatives.

One respondent described the concern:

*"They gave me a prescription for Square company's medicine, and he [pharmacy assistant] goes and brings medicine from another company… but isn't there a difference in quality? This one costs fifty Taka, [while] that one costs five Taka. He will bring the five Taka one. He won't bring the fifty Taka one. So, how will my illness be cured?"*

4.3. *Distance of SSK facility from BPL households*: Location of the SSK facility was reported by respondents as one of the major barriers to low utilisation of SSK services, particularly for those residing at a relatively greater distance from the facility.

A respondent stated:

*“Suppose I am suffering from fever, for this, do I need to go to the designated UpHC spending fifty to one hundred taka [in transportation cost]? The fever would be gone if I take medicine of fifty taka [instead]. Then why would I go to the designated UpHC spending one hundred taka?”*

5. *Unavailability of outpatient care:* Cardholders expressed that the availability of free outpatient care through the SSK card would be beneficial. They frequently needed to visit the designated UpHC for outpatient services, but were often required to purchase medicine themselves. Due to financial constraints, some were unable to afford medications, leaving their treatment incomplete.

Some cardholders, particularly those suffering from minor ailments requiring only outpatient care, reported that they had to opt for hospital admission to access free treatment at the UpHC.

One cardholder expressed the need for outpatient care, stating:

*“It would be a huge benefit if they gave [medicine] without admission. We would benefit greatly if they gave service without admission. If they supplied the medicine in the outpatient department, then we could manage much better.”*

Female cardholders shared that, while admitted, they often visited their homes once a day to manage household chores and cooking. On the other hand, male patients frequently avoided seeking care due to the prospect of prolonged hospitalisation, which would result in income loss. A participant explained why the inpatient-only requirement is a major barrier, especially for those who needed to work:

*“I can't get admitted now, I have work in various places, I will have to leave now, but I'm living with my illness. But even if I go now, they won't give medicine. It will take four days. If I'm admitted for four days, who will earn my four thousand [Taka]? [My] family can't run.”*

*Supply-side challenges*

The implementation of SSK was impeded by a range of significant supply-side challenges, from chronic human resource shortages to the unavailability of medical equipment and medicines. These challenges undermined the capacity of the healthcare facilities to deliver the services promised under the scheme. Thematic analysis of the KIIs identified 6 broad issue areas encompassing 11 distinct supply-side themes, as summarised in Table III.

**Table III.** Supply-side challenges identified through KIIs.

Issue areas	Identified themes	Summary results
Low motivation of service providers	Due to increased workload	Staff perceived SSK duties as an additional project with a heavy documentation burden.
	Due to lack of incentives	Promised performance-based financial incentives for the increased workload were never implemented.
Claim settlement	Irregular processing	Reimbursements were irregular, with delays of several months that disrupted facilities' financial planning.
	Manual claim processing	Reliance on a manual, paper-based system led to frequent errors and processing inefficiencies.
	Documentation issues	Claims were often rejected or delayed due to incomplete documentation and missing signatures.
	Lack of support from the physicians	Physicians were uncooperative with documentation, citing a lack of incentives for the extra administrative work.
Unavailability of outpatient services		The inpatient-only package created patient frustration and conflict, as many sought outpatient care.
Staff retention barriers	Frequent transfers	Frequent promotion and transfer of trained staff created a continuous cycle of vacancies and knowledge loss.
	Recruitment challenges	Strict working hours required by the scheme discouraged new healthcare providers from joining the facility.
Shortage of human resources	Vacant positions	A high number of sanctioned posts for both clinical (e.g., consultants) and administrative staff remained vacant.
	Lack of service providers	The insufficient number of providers limited the facility's capacity to treat all patients.
	Lack of experienced human resource	A shortage of skilled administrative staff and coordinators hampered the verification and collection process.
Unavailability of medical equipment and reagents		Lack of essential diagnostic equipment (e.g., digital X-ray, electrolyte analyser) forced patient referrals.

1. *Low motivation of service providers:* Nine out of ten key informants reported low motivation of service providers towards SSK-related activities due to reasons including increased workload and lack of incentives.

1.1. *Due to increased workload*: It was reported that in the designated UpHC, the staff did not fully cooperate in performing duties related to SSK as they perceived it as an additional burden and responsibility on them that increased their workload.

A key informant described this perception, stating:

*“There is a perception among service providers that they have to do extra work for SSK, more work. There is a perception that working for SSK will increase their workload.”*

Service providers considered SSK as a separate project and felt that they were being forced to participate in it. This feeling grew dissatisfaction among the staffs. However, SSK involvement indeed increased the tasks on all types of hospital staff, for example, senior staff nurses needed to submit regular requisition for unavailable medicines, ensure proper storage of received medicines, distribute medicines as required, and provide medications to admitted patients at the time of discharge. Unlike regular patients, whose medication fulfilment did not require this extensive process, these additional efforts were gradually leading to frustration among staff members.

One informant confirmed that the workload issue extended to the administrative level, showing a systemic problem of staff having divided responsibilities:

*“Those of us who work in the SSK Cell do not only work on SSK; we have many other tasks. This creates a workload issue. There is actually no opportunity to work dedicatedly on SSK; we have to do multiple jobs.”*

1.2. *Due to lack of incentives*: Since the workload increased after the introduction of SSK, the staff generally longed for a financial incentive to compensate that. At the planning phase of the project, a commitment was made to provide financial incentives for the additional work.

A key informant confirmed that a performance-based incentive was part of the original plan but was not approved by the leadership committee:

*“Our concept paper clearly states that we will give an incentive based on performance, but we have not been able to bring it before the steering committee meeting yet. We raised it, but the members of the steering committee are not interested in working with this kind of incentive at this moment.”*

As a result, the service providers, especially those in junior positions, showed reluctance to attend to SSK patients. The staffs often felt unmotivated and often raised questions about why they should perform the non-routine additional tasks imposed by SSK, since the proposed performance-based incentive system was not implemented.

2. *Claim settlement:* Based on information from seven key informants, significant and systemic delays in the claim settlement process were identified as a major supply-side challenge.

2.1. *Irregular processing*: A key challenge was the irregular scheduling of claim settlements, which were not conducted consistently on a monthly basis, despite instructions from MoHFW for timely payments. This led to significant gaps in reimbursement, with due payments for claims, which were submitted two to three months before. The SO also did not provide updated claim data on time, sometimes with delays of two to three months.

A key informant confirmed this challenge, stating:

*“Timely claim settlement is not always possible. This is a manpower problem. The scheme operator is supposed to give us updated claims every month, but they don't provide it on time. Sometimes they delay by one or two months, which creates some problems.”*

2.2. *Manual claim processing*: The claim process was not fully digitalised, which contributed to manual errors and inefficiencies. Informants stated that if the system were digitised, errors would be significantly reduced. Initially, claim files were supposed to be checked randomly at the hospital level, but frequent errors necessitated checking all files.

A key informant detailed this operational burden:

*“Our job here is to check some of them randomly. But initially, we saw that many mistakes were being made, so for that reason, we are checking almost every single file. It's a problem for us to check the hard copies.”*

2.3. *Documentation issues*: Incomplete documentation was a frequent cause of delays. For example, in a C-section case, missing signatures could delay payment processing. The requirement to submit physical hard copies of documents, mandated by government financial regulations, added complexity to the process. Many claim files were returned by the SO due to incomplete documentation. In some cases, facility staff rushed submissions with the assumption of quick processing, only to have them rejected later.

One informant indicated a lack of awareness of facility staff, stating:

*“It was seen that for an investigation during treatment, the relevant order is given, but the supporting paper is missing. The medicine is written on the medicine slip, but the slip itself is missing. This is needed since the bill has to be paid for it. This is perhaps a bit of a lack of awareness on their part.”*

2.4. *Lack of support from the physicians*: A significant factor in processing delays was a lack of support from the physicians. Informants observed that physicians showed little interest in assisting with the required documentation. When asked, they cited the absence of financial incentives for SSK-related work, viewing it as an additional burden.

A key informant reported this directly:

*“When we ask, 'Why are you not preparing the file?' They argue that they do not get any incentive for SSK, and we hear many of them say that SSK is not their job; they consider it extra work.”*

Although a coordinator was assigned to prepare the files, they had to obtain doctors’ signatures and ensure that the forms were completed. Since the physicians were often unwilling to take on these administrative tasks, the coordinator had to repeatedly follow up with them.

3*. Unavailability of outpatient services:* Based on information provided by six key informants, the unavailability of outpatient services in the SSK benefit package presented a major implementation challenge and a significant source of patient dissatisfaction.

A key informant stated:

*“Another big issue is OPD [outpatient department]. OPD [service] is not in the package…* *the benefit package has not been made for it. This is needed. This is really a challenge. A big challenge.”*

This gap in service coverage led to several negative consequences. Patients often travelled long distances, incurring transportation costs, only to find that outpatient services were not available and that they had to return home without receiving care. The initial high volume of SSK patients seeking outdoor care reportedly declined over time as they became discouraged by the lack of coverage. The frustration also led to conflicts at the facilities, with some patients reacting aggressively, misbehaving with staff, and using foul language when they realised their cards would not provide the expected benefits.

4*. Staff retention barriers:* Based on information provided by six key informants, two significant barriers to staff retention were identified.

4.1. *Frequent transfers*: Frequent transfers were a persistent and disruptive issue, creating a continuous cycle of personnel shortages that affected service delivery at the district and upazila levels, as well as within the Health Economics Unit. This problem was reportedly more prevalent among service providers than administrative staff. The programme invested significant effort in training personnel, such as the UHFPOs, for the designated healthcare facilities; however, those trained individuals were often promoted and transferred shortly before or after the programme’s initiation, creating major operational challenges.

A key informant highlighted a specific instance:

*“In two of the designated UpHCs, the respective UHFPOs joined [only] one week before the opening; they know nothing [about SSK]. SSK service began there without them knowing anything about the programme, so these are challenges.”*

Although there was a requirement for doctors to stay at a facility for at least two to three years after receiving training, many left after a few months. Despite recommendations to the Ministry to halt transfers during the pilot phase, staff members actively sought reassignments, sometimes using external influences to secure them.

One key informant explained:

*“We are unable to ensure that a doctor stays here for two or three consecutive years. It is difficult to address this because, according to our job structure, all are transferable jobs. You cannot keep someone permanently if you want to.”*

4.2. **Recruitment* challenges*: The programme faced difficulties in recruiting healthcare providers, as many were hesitant to join the SSK-designated facility. This reluctance was partly attributed to the strict office hour requirements imposed by the SSK programme.

A key informant, involved in SSK service provision, explained this recruitment challenge in detail:

*“Many do not want to come when they hear of the designated UpHC. The reason is that, here, one has to arrive at a specific time and leave at a specific time. Many do not want to follow this rule. That's why whoever is posted here does not want to come to the designated UpHC. This is the biggest problem for me, a huge problem.”*

5. *Shortage of human resources:* Based on information provided by five key informants, a significant shortage of human resources was identified as a critical challenge.

5.1. *Vacant positions*: A primary issue was the high number of vacant positions for both clinical and administrative staff. Key informants confirmed that although all sanctioned posts were supposed to be filled in the piloted facilities, that was not achieved.

A key informant detailed the reality of this shortage:

*“It's a huge problem. every Upazila Health Complex is supposed to have 21 doctors. In comparison, here in my hospital, 4 medical officer posts and 3 consultant posts have been vacant for the past year, and in the sub-centres, 5 doctor posts are vacant. Moreover, a post like Medicine Consultant. if there is no Medicine Consultant in my hospital, I don't know how much service can be provided.”*

Specific consultant posts for medicine, ophthalmology, and orthopaedics remained unfilled. The scale of the shortage was significant, with one informant estimating that for every 10 sanctioned posts, only 2–3 were filled, leading to a total of approximately 25–26 unfilled positions.

Administrative roles were also heavily affected, with one informant stating that these positions remained vacant in larger numbers compared to service provider roles. For example, the accounts department, which was supposed to have four personnel, had only two.

5.2. *Lack of service providers*: The insufficient number of service providers directly impacted patient care. One informant stated that due to the staff shortage, their facility was unable to treat all the patients who visited. The lack of certain technical staff also created operational hurdles; for instance, the absence of a laboratory analyser required that many essential tests for SSK patients be conducted at private facilities, which caused delays and inconvenience.

5.3. *Lack of skilled human resources*: The claim settlement process was hampered by a lack of skilled workers and general human resource shortages. At the upazila level, claims were verified by a team led by the Upazila Health and Family Planning Officers (UHFPO), but shortages in both the accounts department and support teams often delayed this verification.

A key informant described this specific challenge:

*“At the Upazila Health Complex, where the claim is primarily verified. we have a challenge because we have no additional manpower there. The Upazila Health Complex already has a manpower crisis in accounts and other support staff, and on top of that, getting the support needed for this work is often not possible.”*

Collecting the multiple supporting documents required, such as medicine slips and investigation reports, was challenging, especially when tests were conducted at outside facilities. Even when pharmacists were trained to prepare bills, the workload was difficult for one person to handle alone. Additionally, the coordinators who were responsible for these tasks sometimes lacked the necessary expertise to handle them efficiently.

6. *Unavailability of medical equipment and reagents:* Based on information provided by three key informants, a significant challenge to the SSK programme was the unavailability of essential medical equipment and reagents, which impacted diagnostic capabilities. A specific challenge was the outdated X-ray machine, which was supposed to be replaced with a digital version. Despite having all other necessary items in the X-ray room, the new machine was not procured at the time of the interview, leaving the room unused and forcing SSK patients to be referred elsewhere for X-ray investigations.

The limited diagnostic capacity prevented the facility from conducting all required tests, making it necessary to refer patients to the district hospital for further investigations. One informant said:

*“… We do not have serum electrolytes, which is needed for diarrhoeal patients. It is not available in any facilities in the upazila. We need to refer the suspected patients to the district hospital.”*

*Ecosystem challenges*

Table IV presents the ecosystem-level challenges associated with the implementation of the SSK project. It summarises the key administrative and planning constraints that impeded the project's successful execution. These ecosystem challenges were identified by key informants in a manner similar to the supply-side challenges.

**Table IV.** Ecosystem challenges identified through KIIs.

Issue areas	Identified themes	Summary results
Administrative and management issues	Ineffective coordination at the local level	The local implementation committee was dysfunctional due to irregular meetings and internal conflicts.
	Changing representatives from the steering committee	Inconsistent representation on the national steering committee delayed high-level decision-making.
Registration process	Erroneous registration	Ineligible non-BPL households were included, while some eligible BPL households were excluded from the scheme.
	Political influence	The beneficiary list was reportedly influenced by local political leaders, leading to inaccuracies.
	Missing cards	Logistical failures in distribution meant some prepared cards never reached the intended beneficiaries.
Challenges in pharmacy and medicine supply	Due to delayed reimbursement	Payment delays of 2–3 months to pharmacies created financial pressure and hampered the medicine supply.
	Due to long waiting time	Patients faced long waits to receive their prescribed take-home medicines upon discharge.
	At the designated district hospital	The referral hospital lacked a dedicated SSK medicine booth, forcing patients to pay out-of-pocket.
Rigid public finance management rules and regulation		Slow, centralised government procurement rules prevented the timely purchase of essential medical equipment.
Incomprehensive benefit package		The limited list of 70 covered diseases did not satisfy the community's broader health needs, causing dissatisfaction.

1. *Administrative and management issues:* Based on the information provided by five key informants, two significant administrative and management issues were identified.

1.1. *Ineffective coordination at the local level*: The local SSK implementation committee, which was essential for ground-level decision-making, suffered from a lack of coordination. The committee was composed of various local officials to ensure inclusivity. However, the committee did not operate according to its Terms of Reference (ToR), with meetings not being held regularly. The busy schedule of the committee president often delayed meetings, while other government officer members also struggled to allocate sufficient time.

A key informant summarised this problem:

*“The SSK management committees are not functioning according to their TOR. The management committees do not hold regular meetings; they are not available on time. They cannot be properly brought on board.”*

A major source of conflict stemmed from the committee’s organisational structure, in which a senior bureaucrat held only a member position while the chairperson was a locally elected representative. This was perceived as disrespectful by the committee members, resulting in limited cooperation and irregular attendance at meetings. Such hierarchical tension, often characterised as bureaucratic–political conflict, offers a plausible explanation for the local management committee’s inability to operate in accordance with its ToR.

1.2. *Changing representatives from the steering committee*: At a higher level, the national steering committee faced issues of continuity. It functioned like other inter-ministerial committees, where different representatives from the ministry attended each meeting. As a result, project staff had to repeatedly explain the same issues to new representatives before any decisions could be made, causing significant delays in resolving problems and moving forward.

2. *Registration process:* Based on the information provided by four key informants, the beneficiary registration process was undermined by significant challenges, including erroneous targeting, political influence, and logistical issues in card distribution.

2.1. *Erroneous registration*: A primary challenge identified by multiple informants was the erroneous selection of beneficiaries. It was reported that some individuals who did not belong to the BPL population were included in the registration list and received SSK cards, while some genuinely poor and deserving individuals were excluded. One informant, who was also a service provider, noted that while treating patients, it was apparent that some were "relatively solvent" and did not fit the BPL criteria.

As one informant stated:

*“While providing treatment, it seemed to us for a few that they are relatively solvent, and they are not at the BPL level. They might have come under the card's coverage, somehow, by any means. this was a BPL-related challenge for me.”*

2.2. *Political influence*: Two key informants highlighted that the preparation of the BPL beneficiary list was subject to political influence. The list had been developed under successive political administrations, allowing opportunities for partisan involvement in beneficiary selection. This political factor was a likely contributor to the registration errors.

2.3. *Missing cards*: A logistical issue was reported in the final stage of card distribution. In some cases, cards that were distributed through the Union Parishad (local government council) were lost and did not reach the intended recipients, requiring a redistribution effort.

3*. Challenges in pharmacy and medicine supply:* Three key informants identified significant challenges related to the pharmacy and the reliable supply of medicine, which undermined both operational efficiency and patient satisfaction. These issues were primarily driven by delayed reimbursements, operational issues, and service gaps at the referral district hospital.

3.1. *Due to delayed reimbursement*: A recurring theme across multiple interviews was the delay in claim settlements for the pharmacy, which created financial and operational instability. The reimbursement process was characterised as lengthy and complex, involving multiple verification stages whereby bills were first reviewed by the SO and subsequently forwarded to the HEU for final approval and payment. According to informants, this process often took two to three months to complete, creating substantial financial pressure on the contracted pharmacy.

This financial uncertainty had several implications. First, it hindered the efficient management of the SSK pharmacy and the timely repayment of its bills. Second, pharmacies occasionally hesitated to dispense medicines to patients because of pending reimbursements. Third, medicine supply contractors sometimes became unwilling to deliver drugs owing to persistent payment delays.

A key informant detailed this situation:

*“Claim settlement is a long-term process... When a gap of two to three months is created, they [the pharmacy contractor] start hammering us in various ways, pressurising us that if we are not given our money on time, we will stop the medicine supply. This was a big challenge in the beginning and still exists.”*

3.2. *Due to long waiting time*: Delays in the provision of medicine at the point of care were identified as a key source of patient dissatisfaction. Specifically, informants noted that during patient discharge, there were delays in providing the required take-home medicines, which negatively impacted the patient experience.

A key informant from the facility management confirmed this specific issue, stating:

*“There is a delay in giving medicine at the time of patient discharge, for which the patients become displeased. I have to look into that.”*

3.3. *At the designated referral hospital*: A significant service gap was identified at the designated district hospital. No dedicated SSK medicine store existed at the hospital, compelling referred patients to obtain their prescribed medicines from external pharmacies. This posed a significant challenge, as some patients were unable to bear these OOP expenses. Even among those who could afford the medicines, informants reported difficulties in securing reimbursements due to documentation-related problems, resulting in considerable patient dissatisfaction.

A key informant from the SO team described the challenges patients faced when referred to the district hospital:

*"From the patients’ perspective, several challenges were observed. For example, when a patient is referred to the designated referral hospital, we have an SSK booth there, but there is no medicine store. here [at the UpHC], patients do not buy any medicine, but at the designated referral hospital, they have to buy medicine from outside. Often, they do not have the ability to do so. Even when patients managed to buy the medicines, they often faced difficulties submitting reimbursement claims correctly, and delays in claim settlement further prevented timely repayment, leading to considerable dissatisfaction."*

4. *Rigid public finance management rules and regulations:* Based on information from two key informants, the rigid public finance management rules and regulations presented an obstacle to the effective operation of the SSK facilities. These rules prevented health facilities from procuring necessary equipment and planning their budgets according to specific needs, which conflicted with the SSK's core concept of decentralising administrative and financial authority.

The procurement process was centralised and complex. The designated UpHC lacked the authority and budget to purchase equipment directly. Instead, the Central Medical Stores Depot (CMHD), funded by the World Bank, held exclusive purchasing authority. To procure items, the designated UpHC had to submit a list of required equipment to a World Bank web portal for approval. The request was then forwarded to the health ministry for a second approval, before CMHD authorised the purchase.

This multi-tiered approval process was not only time-consuming but also frequently led to the rejection of procurement requests. One informant noted that despite securing the required budget, nearly half of the requested equipment was not approved. A specific example cited was the allocation for a digital X-ray machine, for which procurement approval was denied, thereby preventing its purchase.

A facility manager described the situation:

*“To date, the outdated X-ray machine has yet to be replaced with a digital one. a room with an air conditioner has been ready since last year, but we have not been able to install a digital X-ray machine yet. As a result, the room stays unused, and we are unable to provide the service to the public.”*

5. *Incomprehensive benefit package:* According to two key informants, the limited scope of the benefit package was identified as a key implementation challenge, leading to significant patient dissatisfaction and misunderstanding. Although the benefit package was expanded from the original 50 covered diseases to 70, the challenge persisted because the spectrum of illnesses presented by patients remained inherently unpredictable. When patients sought care for conditions not included among the 70 listed diseases, providers faced difficulties in delivering services under the SSK scheme.

This discrepancy between patient expectations and the scheme’s actual provisions generated considerable dissatisfaction. Patients who were ineligible for treatment under the benefit package often misunderstood the reasons for their exclusion, particularly when they did not receive a full supply of medicines from the facility. Such experiences frequently led patients to pressure staff and question the overall value of the SSK card.

One informant described:

*“When a patient presents with a condition not included among the 70 covered diseases, we are placed in a difficult situation. They often insist, saying, “Why were we given the card if you cannot provide us with treatment under this scheme?”*

## Discussion

Our findings reveal that the SSK project was undermined by a series of interconnected demand-side, supply-side and ecosystem challenges. The evidence suggests the scheme's low utilisation and eventual discontinuation of the pilot from July 2024 were not caused by an isolated issue but rather reflected systemic challenges within the implementation process. These challenges were rooted in a profound disconnect between the programme's high-level ambitions and the on-the-ground realities of both the health system and the communities it was intended to serve.

Our findings suggest that although the SSK scheme provided free inpatient care, it experienced low utilisation among the target population in the first two years. Specifically, approximately 71% of enroled individuals with a reported need for healthcare did not visit the SSK facility within the 90 days preceding the survey. This underutilisation appears to have persisted over time, as a later study by Hasan et al. (2022) found that only 8% of sick individuals in Kalihati upazila sought healthcare from the SSK scheme. In contrast, a larger portion sought care from other medically trained providers (28.2%) or non-medically trained providers (63.8%). These findings contrast with evidence from other countries. Studies in India, Ghana, and Ethiopia, for instance, have shown that similar initiatives significantly increased inpatient healthcare utilisation among beneficiaries (Dalinjong & Laar, 2012; Mebratie et al., 2019; Philip et al., 2016). A primary reason for the low utilisation of the SSK scheme was the prevalence of a knowledge gap among enroled households regarding its services and benefit package. This is highlighted by our finding that of the 83.9% of cardholders who had never used the service, a majority (54.4%) cited not knowing how to use their cards as the main reason for non-use. Furthermore, there was a general lack of awareness about specific benefits, such as coverage for medicines, diagnostic tests, and referrals. This information deficit likely acted as a major barrier, discouraging members from seeking care. This finding is consistent with research from Nigeria, where healthcare providers identified poor patient understanding of scheme benefits and provisions as a significant barrier to participation (Shobiye et al., 2021). Other studies further confirm that a member's knowledge of a scheme affects both healthcare utilisation and dropout rates (Patience et al., 2013; Yusuf et al., 2019). Although the SSK incorporated a comprehensive Information, Education, and Communication (IEC) strategy, it was implemented after the completion of the community survey. As a result, this study could not assess its effectiveness or identify potential implementation gaps.

Our qualitative findings confirmed this lack of awareness among beneficiaries and identified its primary causes, which included limited communication from local leaders and a lack of accurate information among community health workers. However, the FGD results suggested that demand-side challenges extend beyond a mere lack of knowledge. Beneficiaries who attempted to use their SSK cards faced significant barriers, including treatment denial, disrespectful behaviour from service providers, low quality of care, long waiting times, and being required to make OOP payments for services that were supposed to be covered. These challenging experiences created a disincentive for future care-seeking, not only for the individuals involved but also for their communities, as negative word-of-mouth spread. The perception of discrimination, where quality of care was seen to depend on personal connections, further eroded trust in the scheme.

These demand-side challenges identified in the study are not unique to the SSK scheme. Similar demand-side barriers have been widely documented in comparable schemes across other low-and middle-income countries (LMICs). A frequently reported issue is the negative attitude of providers; studies in Ghana, for example, found that rude behaviour and discrimination against insured members were major disincentives to seeking care (Agyepong et al., 2016). A number of studies identified discrimination against scheme members as a significant challenge that hinders scheme enrolment and adversely affects patient satisfaction for such schemes (Alkenbrack et al., 2013; Kyomugisha et al., 2009; Mulupi et al., 2013). Studies have also consistently identified long wait times in the facility as a factor that hinders enrolment and increases member dropout from insurance schemes (Alkenbrack et al., 2013; Mulupi et al., 2013; Onwujekwe et al., 2009). Similarly, the practice of requiring OOP payments for services and medicines that were supposedly covered by the scheme was a common source of beneficiary discontent in a similar scheme in Ghana (Agyepong et al., 2016). Furthermore, perceptions of poor quality of care were a key barrier. In the SSK scheme, cardholders cited ineffective treatments and the frequent use of a limited range of common drugs as primary reasons for their dissatisfaction. This finding is consistent with research in other contexts, such as in Lao PDR, scheme members reported receiving low-quality drugs and questioned the diagnostic skills of healthcare staff (Alkenbrack et al., 2013). As numerous studies confirm, poor quality of care in its various forms was a key factor that negatively affects enrolment and service utilisation, thereby undermining a programme's success (Basaza et al., 2008; Mulupi et al., 2013; Onwujekwe et al., 2009).

On the supply side, our study identified significant barriers related to human resource management. Key informants consistently cited provider demotivation, staff shortages, and poor retention as the primary challenges. The low motivation among service providers was attributed to two main factors: (i) a substantially increased workload; (ii) a lack of financial incentives. Staff felt overburdened by the dual demands of treating SSK patients and other non-member patients at the same time. Although the SSK's design included performance-based incentives to compensate for this extra work, the policy was not implemented, leaving staff to work overtime without additional pay and fostering low morale. The challenge of an increased provider workload has been reported in similar programmes in Ethiopia and Ghana, where higher patient loads negatively affected provider behaviour and hampered the quality of care. While an increased workload may be a common consequence of such schemes, the complete absence of a system to compensate for these additional efforts was a critical finding in the SSK's case. The importance of establishing provider incentives was highlighted in the broader literature, with a systematic review identifying them as crucial for ensuring provider commitment and the success of CBHI schemes in LMICs (Dalinjong & Laar, 2012; Fadlallah et al., 2018; Hussien et al., 2022; Witter et al., 2013). The staff shortages identified in the SSK scheme stemmed primarily from challenges in filling the sanctioned positions. The challenge of an inadequate workforce is a well-documented barrier that constrains the implementation of similar schemes elsewhere. Studies in Tanzania and Nigeria, for instance, found that staff shortages negatively affected the quality of care and patient satisfaction, thereby hindering programme success (Kamuzora & Gilson, 2007; Onwujekwe et al., 2009; Shobiye et al., 2021).

One of the unique implementation challenges that the SSK scheme faced was the staff retention barriers. A primary barrier to retention was the SSK management's lack of authority over personnel decisions. Since the staff postings and transfers were controlled centrally by the Directorate General of Health Services (DGHS), the local scheme could not ensure the retention of its trained providers. This issue was compounded by recruitment difficulties, as the facility's strict work schedule was reportedly unattractive to new providers when compared to other non-designated UpHCs. The finding that centralised control over personnel can undermine local operations is consistent with evidence from other contexts; a study of Ghana's health insurance scheme, for instance, reported that the performance of district offices was negatively impacted by similar staffing challenges rooted in the central allocation of personnel (Agyepong et al., 2016).

In addition to the issues with human resources, informants identified irregular claim settlements as a major operational failure of the SSK scheme that hindered both service quality and programme operations. Delays in reimbursing providers and members are a commonly cited implementation challenge for CBHI schemes. Studies from Ghana, Nigeria, and Ethiopia consistently identified slow and irregular claim settlements as a major challenge. In Ghana, reimbursement delays were a primary concern for both purchasers and providers, with reports of providers not being paid for over six months, far exceeding the legally mandated four-week period (Agyepong et al., 2016; Dalinjong & Laar, 2012). These financial delays have tangible consequences for healthcare delivery. As observed in Ghana, providers facing long reimbursement delays were often unable to procure necessary drugs and medical supplies, disrupting the smooth operation of their facilities. In some cases, funding delays led facilities to withhold free services from scheme members altogether (Dalinjong & Laar, 2012; Witter et al., 2013). A critical consequence of these delays was the negative impact on provider behaviour and patient trust. These reimbursement issues affected provider responsiveness, reduced patient satisfaction, and discouraged enrolment in the scheme (Agyepong et al., 2016; Hussien et al., 2022; Okunna et al., 2022; Shobiye et al., 2021). Unavailability of medical equipment and reagents was another supply side barrier identified by the key informants. This shortage not only undermined service provision but also reflected the consequences of restrictive and centralised public finance management. Similar challenges have been documented in other settings; for example, a study from Ghana reported that centralised control over funding, staffing, and logistics directly hampered the performance of district level scheme offices (Agyepong et al., 2016).

A critical design limitation of the SSK scheme, identified by both service providers and beneficiaries, was the exclusion of outpatient services from its benefit package. This omission was a primary barrier to the programme's success, a finding that aligns with research highlighting the importance of comprehensive coverage. The literature confirms that including outpatient care is crucial for the viability of such schemes. A study conducted in rural China reported that outpatient coverage positively affected enrolment, accessibility, and service utilisation (Hao et al., 2010). Similarly, a study based in Kenya indicated that communities preferred scheme that covered both inpatient and outpatient services, including all associated costs for treatment, drugs, and transport (Mulupi et al., 2013).

Finally, our analysis shows that the implementation barriers were not confined to the demand side and supply side but were embedded within a wider ecosystem that connects policy formulation to front line execution. Weak governance and coordination between the national steering committee and local management committees, political influence and logistical failures in beneficiary registration, rigid centrally controlled public financial rules, and fragmented pharmacy and medicine supply arrangements collectively shaped who was enroled, what services could be delivered, and how quickly problems could be resolved. In addition, the design of the benefit package was perceived to be narrow relative to the community's health needs. The problem of a limited benefit package has been documented in literature, with a systematic review identifying it as a major reason for non-renewal of membership and a study from Kenya linking it to both low enrolment and high dropout rates (Hussien & Azage, 2021; Mulupi et al., 2013).

### Policy recommendations

Based on the identified supply-side, demand-side and ecosystem challenges, several policy actions are recommended to enhance the effectiveness and sustainability of the SSK scheme (see Appendix 1). Strengthening institutional governance through the establishment of an autonomous National Health Security Office under the Ministry of Health, with authority over financial management, service delivery, and accreditation, would ensure accountability and long-term sustainability. Addressing the persistent human resource shortages requires the recruitment and retention of dedicated SSK personnel at both district and upazila levels, coupled with performance-based incentives to improve provider motivation and service quality. Digitisation and decentralisation of claim management and reimbursement processes are essential to reduce delays and enhance transparency. Greater managerial flexibility in procurement and financial allocation would also improve the timely availability of essential medical supplies and equipment.

To enhance beneficiaries' understanding of the scheme, sustained community awareness initiatives using locally appropriate approaches, such as household visits, yard meetings, and school-based promotion, should be prioritised. Expanding the benefit package to include outpatient and diagnostic services would mitigate a major source of dissatisfaction and promote higher service utilisation, while annual updates of BPL lists using participatory and data-driven methods would enhance targeting accuracy. To advance equity and financial sustainability, the scheme should progressively extend coverage to the APL population through voluntary enrolment, supported by improved outpatient services, strengthened facility infrastructure, and robust quality assurance mechanisms. Furthermore, integrating private healthcare facilities into the SSK network under a regulated pricing and accreditation framework could expand service capacity and ensure continuity of care when public facilities reach capacity. Collectively, these policy measures can help address the implementation challenges identified in this study and serve as actionable guidance for the design and strengthening of any similar health protection scheme implemented in Bangladesh or comparable low-resource settings.

### Limitations

A primary limitation of this study is that the data were collected in 2018. However, the recent operational pause of the SSK scheme makes this analysis both timely and highly relevant. This pause provides a critical opportunity to look back and diagnose the deep-rooted implementation challenges that hindered the programme's success. The fact that the scheme remained in a pilot phase for eight years without achieving national or divisional adaptation is a clear indicator of these persistent obstacles, which this study helps to elucidate. Another possible shortcoming of this study is that the design of the community survey is cross-sectional in nature. However, this is the first study that examines the demand side and supply side challenges of SSK implementation using a mixed-methods approach. There was also a possibility of recall bias as health service utilisation data were collected using a self-reported questionnaire in the community survey. A similar study used recall periods of 12 months for collecting similar data (Alkenbrack et al., 2013; Mebratie et al., 2019; Umeh & Feeley, 2017). We used a 90-day recall period to reduce the possibility of recall bias. Finally, since the household head served as the primary respondent in the community survey, the responses may not fully capture the experiences of all individual household members.

## Conclusion

This study identified critical implementation challenges constraining a government-funded health protection scheme for the BPL population in Bangladesh. The identified challenges largely hindered this promising scheme from effectively contributing to Bangladesh's goal of achieving UHC. A thorough resolution of these supply-side, demand-side, and ecosystem barriers is a critical prerequisite for the successful design and implementation of any similar health protection initiative, both in Bangladesh and in other low-income settings.

## Supplementary Material

Supplementary material.docx Supplementary material.docx

## Data Availability

The data that support the findings of this study are available from the corresponding author, SA, upon reasonable request.

## Appendix 1

**Table A1.** Policy recommendations to address the implementation challenges of the SSK scheme.

SN	Policy recommendations	Targeted challenges	Expected impact
1	Establish a National Health Security Office (NHSO) under the Ministry of Health by transforming the Health Economics Unit (HEU) into an independent authority for social health protection, with legal backing through the Health Protection Act. The NHSO should integrate financial, service delivery, and accreditation functions and oversee both public and private facilities, including claim settlement management.	Weak institutional governance; Inefficient management and claim processing	Strengthened institutional coordination and accountability to ensure continuity and sustainability of the scheme, streamline claim settlements, improve transparency, enable nationwide scale-up, and institutionalise social health protection within the UHC framework.
2	Expand the SSK benefit package to include outpatient and diagnostic services alongside inpatient care to meet the population’s healthcare needs	Unavailability of outpatient care	Expanded service coverage to increase patient flow and satisfaction, reduce unnecessary admissions, and prevent income loss from hospitalisation.
3	Recruit and retain dedicated SSK personnel at district and upazila levels, including medical personnels, IT specialists, accounts officers, and support staff for documentation and claim processing.	Shortage of human resources	Reinforced institutional capacity and operational efficiency to reduce claim processing delays, improve accountability, enhance service quality, and enable continuous monitoring and timely resolution of administrative and financial issues.
4	Introduce performance-based financial and non-financial incentive mechanisms for service providers by linking rewards to workload, professionalism, and institutional performance.	Staff retention barriers; Low motivation of service providers	Enhanced provider motivation and job satisfaction, leading to improved service quality, better performance, and retention.
5	Digitise and decentralise claim management and reimbursement processes by introducing online documentation, electronic record-keeping, and district-level settlement units to minimise manual errors and accelerate verification and approval	Claim settlement	Expected to improve administrative efficiency, reduce reimbursement delays, maintain regular medicine supply, and strengthen financial accountability.
6	Fill all vacant and sanctioned doctor positions to ensure adequate staffing and uninterrupted service delivery	Shortage of human resources	Increased service availability to ensure full coverage, improved patient access and continuity of care, and enhanced efficiency and credibility
7	Revise public financial management regulations to allow greater flexibility for SSK facilities in procurement and resource allocation based on demonstrated efficiency and cost-effectiveness.	Rigid public finance management rules and regulation	Improved availability of essential equipment and reagents to ensure uninterrupted diagnostic services, reduce referral delays, and enhance the overall operational efficiency
8	Update and verify the Below-Poverty-Line (BPL) beneficiary list annually using participatory and data-driven methods to ensure accurate inclusion and exclusion of households	Erroneous registration process	Improved targeting accuracy to ensure equitable access, prevent misuse of benefits, and enhance credibility
9	Expand SSK coverage to include the above-poverty-line (APL) population through voluntary enrolment by upgrading outpatient services, facility infrastructure, and service quality to ensure equitable participation and financial sustainability.	Sustainability	Increased population coverage to establish cross-subsidy within the scheme, improve financial sustainability, enhance trust in public healthcare, and promote universal access
10	Implement community awareness campaigns using locally appropriate methods such as miking, yard meetings, and household visits by community health workers to disseminate information on eligibility, covered diseases, and service procedures.	Poor knowledge of cardholders	Improved awareness and understanding of the SSK scheme, leading to increased utilisation of entitled benefits, reduced misinformation among beneficiaries and enhanced community trust and health-seeking behaviour.
11	Ensure round-the-clock availability of doctors and support staff to provide timely care for emergency and inpatient cases under the SSK programme	Dissatisfaction with SSK services	Improved responsiveness and quality of care to strengthen patient trust, reduce waiting time, and enhance the overall reliability
12	Ensure regular attendance and active participation of members in monthly SSK implementation committee meetings and assign a dedicated ministry representative to all steering committee sessions to address implementation challenges promptly	Administrative and management issues	Improved coordination and decision-making to accelerate claim settlements, enhance accountability, and strengthen the overall governance and responsiveness of the programme
13	Integrate private healthcare facilities into the SSK network through a regulated pricing and accreditation system to expand service capacity and ensure continuity of care when public facilities reach capacity.	Sustainability	Expanded service availability to increase treatment capacity, reduce patient overflow from public facilities, and promote public–private collaboration for sustainable service delivery.
14	Implement monthly school-based awareness programmes where students convey key health protection messages to their families and communities.	Poor knowledge of cardholders	Increased community awareness and participation to strengthen intergenerational knowledge transfer and promote preventive health-seeking behaviour.

**Glossary**

**Table:**

Term	Definition
Below-Poverty-Line (BPL) Population	The segment of the population whose income falls below a nationally defined poverty threshold.
Benefit Package	The specific set of healthcare services, treatments, medicines, and diagnostic tests that are covered by a health insurance or protection scheme.
Catastrophic Health Expenditure (CHE)	Healthcare spending that exceeds a certain threshold of a household's income (e.g., 10% or 25%), potentially forcing the household into poverty.
Claim Settlement	The process through which healthcare providers are reimbursed by the insurance scheme operator for the covered services they have delivered to an enroled member. This involves submitting documentation and receiving payment.
Out-of-Pocket (OOP) Expenditure	Direct payments made by individuals for healthcare services at the time of use, such as payments for doctor's fees, medicines, or hospital stays, that are not reimbursed by any third party.
Scheme Operator	The Scheme Operator is a contracted organisation responsible for managing operational activities of a health protection scheme, including beneficiary enrolment, claim processing, and coordination of service delivery at designated health facilities.
Shasthyo Suroksha Karmasuchi (SSK)	Government-funded social health protection scheme in Bangladesh that is the subject of the study.
Social Health Protection Scheme	A system designed to provide financial access to healthcare for a population, typically funded through taxes or social security contributions, to protect households from high and unpredictable medical costs.
Universal Health Coverage (UHC)	A health system goal where all individuals and communities can access the full spectrum of essential, quality health services they need—from health promotion to prevention, treatment, and rehabilitation—without suffering financial hardship.
Upazila	An administrative sub-district in Bangladesh. It is the primary unit for local governance and public service delivery, including healthcare.
Upazila Health Complex (UpHC)	A government-run public hospital at the sub-district (Upazila) level in Bangladesh, serving as a primary referral centre and providing both inpatient and outpatient care for the local population.
