# Supplementary material for: Implementation challenges for achieving universal health coverage through social health protection scheme: what can we learn from Bangladesh?
Source: Int J Qual Stud Health Well-being. 2026 Feb 7;21(1):2623094. doi: 10.1080/17482631.2026.2623094 (PMC12885007; doi:10.1080/17482631.2026.2623094)
Supplement: Supplementary material.docx [file ZQHW_A_2623094_SM8540.docx]

**Supplementary material**

This document presents supplementary materials for the study titled, “**Implementation challenges for achieving universal health coverage through social health protection scheme: what can we learn from Bangladesh?**”. It includes: (1) key informant interview guidelines for different stakeholders, (2) focus group discussion guidelines for beneficiaries, and (3) the community survey questionnaire used for data collection.

**Contents**

[Material 1: Key Informant Interview (KII) guidelines 2](#_Toc219415725)

[Supplement 1a: KII guideline for Service provider personnel 2](#_Toc219415726)

[Supplement 1b: KII guideline for insurance personnel 3](#_Toc219415727)

[Supplement 1c: KII guideline for Health Economics Unit personnel 4](#_Toc219415728)

[Material 2: Focus Group Discussion (FGD) guideline for beneficiaries 5](#_Toc219415729)

[Material 3: Community survey questionnaire 6](#_Toc219415730)

# Material 1: Key Informant Interview (KII) guidelines

## Supplement 1a: KII guideline for Service provider personnel

1. **Identification**
2. Date of interview:
3. Name of service center:
4. Name of interviewee:
5. **General Information**

Tell us about yourself

**Probe:**

1. Personal details: Name, Age, Education
2. Current professional status: Duration of current profession, previous professional experience
3. Your role in the Shasthyo Suroksha Karmasuchi (SSK) project

**3. Challenges related to SSK project implementation**

**A. What is your opinion on the activities of the SSK project? What do you think are the obstacles to implementing the SSK project?**

**Probe:**

1. What is your responsibility in the SSK project?
2. What kinds of problems have you faced while performing your duties for this project? How are these problems creating obstacles to your work?
3. How does working on this project interfere with your other professional responsibilities?
4. Is the work you do easy for you? Do you receive help from anyone else while performing ancillary duties related to the project?
5. While carrying out the additional responsibilities related to the project, have you received any kind of assistance from anyone? What types of support from others would make it easier for you to perform these project-related additional tasks and increase the efficiency of your work?
6. What kind of barriers do you think poor people face in enrolling in this project?
7. What kind of obstacles do you think will arise if this project is implemented on a large scale across the country?

**B. How can these obstacles be overcome? What are your suggestions?**

**Probe:**

1. What steps can be taken to eliminate the obstacles you have mentioned?
2. In your opinion, what other benefits could be added to the project for better results?
3. How can officials associated with this project be encouraged to maintain professional conduct while performing their duties?
4. To manage this project successfully, what do you think the future strategy should be?
5. In what ways can the obstacles you foresee for a nationwide project scale-up be removed?

**Instructions for the interviewer:**

Summarize the main comments from the interview and ask the interviewee if they have anything else to add. Finally, thank the interviewee and conclude the interview.

## Supplement 1b: KII guideline for insurance personnel

**1. Identification**

1. Date of interview:
2. Name of service center:
3. Name of interviewee:

**2. General Information**

Tell us about yourself

**Probe**

1. Personal details: Name, Age, Education
2. Current professional status: Duration of current profession, previous professional experience
3. Your role in the Shasthyo Suroksha Karmasuchi (SSK) project

**3. Challenges related to SSK project implementation**

**A. What is your opinion on the activities of the SSK project? What do you think are the obstacles to implementing the SSK project?**

**Probe:**

1. What is your responsibility in the SSK project?
2. What kinds of problems have you faced while performing your duties for this project? How are these problems creating obstacles to your work?
3. How does working on this project interfere with your other professional responsibilities?
4. Is the work you do easy for you? Do you receive help from anyone else while performing ancillary duties related to the project?
5. While carrying out the additional responsibilities related to the project, have you received any kind of assistance from anyone? What types of support from others would make it easier for you to perform these project-related additional tasks and increase the efficiency of your work?
6. What kind of barriers do you think poor people face in enrolling in this project?
7. According to SSK project guidelines, what kind of difficulties do you face in identifying eligible individuals and in the associated activities of providing them with SSK cards?
8. What types of problems do you face in delivering service-related information to the Health Economics Unit and in settling financial claims for those services?
9. What kind of obstacles do you think will arise if this project is implemented on a large scale across the country?

**B. How can these obstacles be overcome? What are your suggestions?**

Probe

1. What steps can be taken to eliminate the obstacles you have mentioned?
2. In your opinion, what other benefits could be added to the project for better results?
3. How can officials associated with this project be encouraged to maintain professional conduct while performing their duties?
4. In your opinion, what should the future course of action or strategy be to successfully carry out this project?
5. How do you think the problems faced in identifying eligible individuals for SSK benefits can be resolved?
6. How can the problems you mentioned in settling service-related financial claims be removed, and what measures do you think would bring more efficiency to the claim settlement process?
7. In what ways can the obstacles you foresee for a nationwide project scale-up be removed?

**Instructions for the interviewer:**

Summarize the main comments from the interview and ask the interviewee if they have anything else to add. Finally, thank the interviewee and conclude the interview.

## Supplement 1c: KII guideline for Health Economics Unit personnel

**1. Identification**

1. Date of interview:
2. Name of service center:
3. Name of interviewee:

**2. General Information**

Tell us about yourself

**Probe**

1. Personal details: Name, Age, Education
2. Current professional status: Duration of current profession, previous professional experience
3. Your role in the Shasthyo Suroksha Karmasuchi (SSK) project

**3. Challenges related to SSK project implementation**

**A. What is your opinion on the activities of the SSK project? What do you think are the obstacles to implementing the SSK project?**

**Probe:**

1. What is your responsibility in the SSK project?
2. What kinds of problems have you faced while performing your duties for this project? How are these problems creating obstacles to your work?
3. How does working on this project interfere with your other professional responsibilities?
4. Is the work you do easy for you? Do you receive help from anyone else while performing ancillary duties related to the project?
5. While carrying out the additional responsibilities related to the project, have you received any kind of assistance from anyone? What types of support from others would make it easier for you to perform these project-related additional tasks and increase the efficiency of your work?
6. What kind of barriers do you think poor people face in enrolling in this project?
7. According to SSK project guidelines, what kind of difficulties do you face in identifying eligible individuals and in the associated activities of providing them with SSK cards?
8. In the current process, what kinds of problems do you face when verifying the authenticity of service-related documents from service centers and processing financial claims accordingly?
9. What kind of barriers do you think poor people face when trying to enroll in this project?

**B.** How can the obstacles be addressed? What is your advice on this matter?

**Probe:**

1. What steps can be taken to eliminate the obstacles you have mentioned?
2. In your opinion, what other benefits could be added to the project for better results?
3. How can officials associated with this project be encouraged to maintain professional conduct while performing their duties?
4. To successfully manage this project, what do you think the future work strategy should be?
5. How can the limitations/disadvantages you mentioned in the current financial claim payment process be resolved? Do you think a new method for verifying documents and paying claims could bring more efficiency to these activities?
6. In what ways can the obstacles you anticipate in implementing the project on a large scale across the country be removed?

**Instructions for the interviewer:**

Summarize the main comments from the interview and ask the interviewee if they have anything else to add. Finally, thank the interviewee and conclude the interview.

# Material 2: Focus Group Discussion (FGD) guideline for beneficiaries

**Objective(s) of FGD**

1. Knowledge of SSK services.
2. Satisfaction on the SSK services
3. Barriers to utilize services under the scheme
4. Effectiveness of IEC strategies.

**Guiding Questions**

1. How did you or your community first learn about the SSK scheme?

**Probe**: Who informed you about it, through which channels did you come to know, which means were most effective for you, and how could the information be better communicated for easier understanding?

1. What do you know about the SSK scheme?

**Probe**: Knowledge about SSK, how it functions, and the types of benefits or services it provides.

1. How were you enrolled as an SSK cardholder?

**Probe**: Who conducted the registration and what was the registration process like?

1. What usually happens when you arrive at the health facility?

**Probe**: Admission procedures, initiation of services, and behaviour of healthcare providers.

1. What types of services do you receive under the SSK scheme?

**Probe**: Number of diseases covered, specific services included (such as medicines, diagnostics, referrals, etc.).

1. Where do most people in your community seek treatment when they fall ill?
2. How do the service providers treat you?

**Probe**: Behaviour of SSK booth staff, nurses, doctors, and other employees—whether they communicate respectfully during registration, discuss your problems properly, interact with a welcoming attitude, and give sufficient time to patients.

1. Are you receiving the services and benefits that were promised at the beginning of the SSK scheme?

**Probe**: Availability of diagnostic services, adequacy of medicines in the pharmacy, and transport arrangements for referrals.

1. Do you have any feedback or suggestions regarding the SSK scheme?

**Probe**: What could be done to improve it, ensure better service delivery, or enhance the process of identifying genuine beneficiaries.

1. Have you faced any difficulties while receiving services under SSK?

**Probe**: Nature of challenges encountered and your opinions regarding them.

1. What additional measures could be taken to increase public awareness and participation in the SSK scheme?

# Material 3: Community survey questionnaire

[Note: This was the latest online version of the questionnaire. Please see next page]

**Study title: A study to systematically document the implementation related challenges of *Shasthyo Suroksha Karmasuchi* Health Protection Scheme of the Government of Bangladesh**

**Principal Investigator PI:** _________

**Organization:** ________

**Purpose of this research**

Greetings! I am Name of the interviewer ----------------- from iccdr,b, an international health research organization. To identify the implementation related challenges of *Shasthyo Suroksha Karmasuchi* health protection scheme of the government, we are conducting a research in this area**.** The purpose of this research is to assess whether the beneficiary under this scheme are identified according to the predefined criterion and provide feedback to the program implementer identifying the implementation related challenges of the scheme. For this purpose, we will ask you about your knowledge of the scheme, your household income, asset, healthcare utilization and related expenses.

***Why did we select you?***

Since this project is being conducted in this area and you are randomly selected for this study, we have considered you as a respondent of this study.

***Method***

If you agree to participate in this research study, it would involve an interview lasting about 30 minutes. In this interview, I shall ask you about your income and your time involvement and expenditure to receive this service. You can choose your comfortable place for interview. If you agree I can start interview now or I can come again at your convenient time.

***Privacy, anonymity and confidentiality***

We are assuring you that information given by you will be kept strictly confidential. We also want to assure you that all records of the interviews will be kept in a safe and secure place for five years and will not be used for any other purposes than the study. We ensure you that your name and other identity will not be exposed while the research results will be published. So it will not be possible to trace the answers back to you.

***Future use of information:***

Information provided by you will be used for this research only and your name will not be exposed when the research results will be published.

***Risk***

There are no physical and social risks related to your participation in this study and if you refuse to participate in this study, you and your family will not face any risk.

***Benefits***

You will not be directly benefited by participating in this study. However**,** this information will help to improve the current project, efficiency and services.

***Freedom of not to participate and withdraw*:**

You are absolutely free to either participate or not participate in the study. You are free not to answer any question if you wish. Even you are free to withdraw at any point of the interview.

***Compensation***

The study is unable to provide any financial compensation to you.

If you have any queries regarding the study, you are free to ask the interviewer. You can also contact the principal investigator of this study or IRB coordinator at the address given below.

| Are you agree to participate in this study | **Yes No** |  |
| --- | --- | --- |
| Signature or left thumb impression of participant | **______________________** | **Date________________** |
| Signature of the supervisor of the survey | **______________________** | **Date________________** |
| Signature of the Interviewer | **_____________________** | **Date________________** |

| **Section 1: Identification** |
| --- |
| **Questionnaire code:** |
| **Name of the respondent ______________________________________________________________**  **Is the respondent a household head? ......1. Yes ...... 2. No**  **Address of the respondents-For SSK members write as mentioned in the card: __________________________________________________________**  ____________________________________________________________________________  **Union: \|___\|___\| Code-1**  **Mobile number: \|___\|___\|___\|___\|___\|___\|___\|___\|___\|___\|___\|**  **Phone number: Yours 1 Not yours 2 If others, Please mention the relationship with the person ___________________________** |
| Is the household under SSK services? 🞏 1. Yes 🞏 2. No |
| Code of the interviewer: \|___\|___\|  Interview starting time: \|___\|___\|: \|___\|___\| 🞏 am/ 🞏 pm Interview end time: \|___\|___\|: \|___\|___\| 🞏 am/ 🞏 pm Date of the interview: \|___\|___\|:\|___\|___\|:\|___\|___\|___\|___\| Day/Months/Year  Record GPS location: |

Code-1

| 1.Kokodhora | 8.Narandia |
| --- | --- |
| 2.Gohaliabari | 9.Paikora |
| 3.Doshkia | 10.Parokhi |
| 4.Durgapur | 11.Bolla |
| 5.Nagbari | 12. Bangra |
| 6.Birbasinda | 13.Solla |
| 7.Sohodebpur | 14.Alenga municipality |

| **Section 2: Information on household members** | | | | | | | | | | | |
| --- | --- | --- | --- | --- | --- | --- | --- | --- | --- | --- | --- |
| C1 | C2 | C3 | C4 | | C5 | C6 | C7 | C8 | C9 | C10 | 11 C11 |
| Member code | Name Short name | Relationship with the household head | Gender | | Age-in integars | Marital status | Educational qualification of the member in years If doesn’t have education write “0” | Main occupation | Does this member earn regularly? Yes 1 No 2 Not-appilcable 99] | How many days the memebr worked in the last 30 days | Earnings of the last 30 days [In taka] |
|  |  | \| 1 \| Self \| \| --- \| --- \| \| 2 \| Husband/wife \| \| 3 \| Father/Mother \| \| 4 \| Son/daughter \| \| 5 \| Father-in-law / mother-in-law \| \| 6 \| Brother/Sister \| \| 7 \| Son-in-law / daughter-in-law \| \| 8 \| Grandson/Grand- daughter \| \| 9 \| Relatives \| \| 10 \| Adopted children \| \| 11 \| Uncle/aunt \| \| 12 \| Grandfather/grandmother \| \| 13 \| Relation with who are not relatives \| \| 14 \| Others Specify \| | 1 | Male |  | \| 1 \| Married \| \| --- \| --- \| \| 2 \| Single \| \| 3 \| Divoreced/divorcee \| \| 4 \| Separated \| \| 5 \| Destitute \| \| 6 \| Others- Please specify \| |  | \| 1 \| Agriculture \| 16 \| Worker \| \| --- \| --- \| --- \| --- \| \| 2 \| Housewife \| 17 \| Fisherman/oarsman/Fish farming \| \| 3 \| Daylaborer \| 18 \| Handycraft \| \| 4 \| Rishkaw/Van driver \| 19 \| Garments worker \| \| 5 \| CNG/Auto driver \| 20 \| Reastauratn/worker \| \| 6 \| Maid \| 21 \| Mechanics \| \| 7 \| Fromal Job \| 22 \| Salesman \| \| 8 \| Shopkeeper \| 23 \| Barber \| \| 9 \| Begger \| 24 \| Tailor \| \| 10 \| Unemployed \| 25 \| Teacher \| \| 11 \| Student \| 26 \| Mobile business \| \| 12 \| old \| 27 \| Not applicable \| \| 13 \| Child \| 28 \| Disabled \| \| 14 \| Farming \| 29 \| Others-specify \| \| 15 \| Sharecropping \|  \| \| |  |  |  |
|  |  |  | 2 | Female |  |  |  |  |  |  |  |
|  |  |  |  | |  |  |  |  |  |  |  |
|  |  |  |  |  |  |  |  |  |  |  |  |
|  |  |  |  | |  |  |  |  |  |  |  |
|  |  |  |  | |  |  |  |  |  |  |  |
|  |  |  |  | |  |  |  |  |  |  |  |
|  |  |  |  | |  |  |  |  |  |  |  |
|  |  |  |  | |  |  |  |  |  |  |  |
|  |  |  |  | |  |  |  |  |  |  |  |
|  |  |  |  | |  |  |  |  |  |  |  |
|  |  |  |  | |  |  |  |  |  |  |  |
|  |  |  |  | |  |  |  |  |  |  |  |

| **Section 3: Information on household** | | | | | | | | | | | | | | | | | | | | |
| --- | --- | --- | --- | --- | --- | --- | --- | --- | --- | --- | --- | --- | --- | --- | --- | --- | --- | --- | --- | --- |
| **Part 3A: Living place and land related information** | | | | | | | | | | | | | | | | | | | | |
| 1 | Are you the owner of the homesteadyou used to live? | | | | 1. Yes → go question no 3 | | | | | | | | | 2. No | | | |  | | |
| 2 | Who is the owner of the homestead? | | | | 1. Others land  2. Roadside/govt. land  3. Rented  (Please mention the area if it is rented)-----sq.ft | | | | | | | | | | | | |  |  |  |
| 3 | How was the living condition of the household? According to the interviwer | | | | 1. Clean 2. Average 3. Dirty 4. Very dirty | | | | | | | |  | | | | |  | | |
| 4 | Do you have any other land except the homestead? | | | | 1.Yes | | | | | | | | 2. No | | | | |  |  |  |
| 5 | Write the amount of land/homestead area | | | | Homestead with pond and pool area:  Paki :_______Decimal:_______ = Decimal\|___\|___\|___\|___\|  Agricultural land  Paki:_______ Decimal: _______ = Decimal\|___\|___\|___\|___\| | | | | | | | | | | | | | | | |
| 6 | How many rooms are there in this household? | | | | Number \|___\| | | | | | | | | | | | | | | | |
| 7 | What kind of materials was used to make the main room of the household to be verified? | | | | 1. Pucca / semi-Pucca 2. Tin 3. Tin and bamboo 4. Tin and others 5. Bamboo and others 6. Soil 7. wood 8. ceramics/ tiles 9. Others | | | | | | | | | | \|  \| Roof \| Wall \| Floor \| \| --- \| --- \| --- \| --- \| \| Serial no \|  \|  \|  \| \|  \|  \|  \|  \| | | | | | |
| 8 | Are these goods / assets available at your household? (More than one answer is acceptable?) | | | | No. | | | | | goods / assets | | | | | | | | | **Yes** | **No** |
|  |  |  |  |  |  | | | | | Cow/goat | | | | | | | | | 1 | 2 |
|  |  |  |  |  |  | | | | | Fishing net | | | | | | | | | 1 | 2 |
|  |  |  |  |  |  | | | | | duck/Chicken | | | | | | | | | 1 | 2 |
|  |  |  |  |  |  | | | | | Grocery | | | | | | | | | 1 | 2 |
|  |  |  |  |  |  | | | | | Riksha/Van/ Auto Riksha | | | | | | | | | 1 | 2 |
|  |  |  |  |  |  | | | | | Agticultural instruments | | | | | | | | | 1 | 2 |
|  |  |  |  |  |  | | | | | Boat | | | | | | | | | 1 | 2 |
|  |  |  |  |  |  | | | | | Bed | | | | | | | | | 1 | 2 |
|  |  |  |  |  |  | | | | | blanket | | | | | | | | | 1 | 2 |
|  |  |  |  |  |  | | | | | Quilt | | | | | | | | | 1 | 2 |
|  |  |  |  |  |  | | | | | Hurricane | | | | | | | | | 1 | 2 |
|  |  |  |  |  |  | | | | | Chair/ Table | | | | | | | | | 1 | 2 |
|  |  |  |  |  |  | | | | | Dining Table | | | | | | | | | 1 | 2 |
|  |  |  |  |  |  | | | | | Amirah / Showcase | | | | | | | | | 1 | 2 |
|  |  |  |  |  |  | | | | | Sofa Set | | | | | | | | | 1 | 2 |
|  |  |  |  |  |  | | | | | Television | | | | | | | | | 1 | 2 |
|  |  |  |  |  |  | | | | | Radio/ Tap Recorder | | | | | | | | | 1 | 2 |
|  |  |  |  |  |  | | | | | Clock/wall clock | | | | | | | | | 1 | 2 |
|  |  |  |  |  |  | | | | | Telephone/ Mobile Phone | | | | | | | | | 1 | 2 |
|  |  |  |  |  |  | | | | | Bicycle | | | | | | | | | 1 | 2 |
|  |  |  |  |  |  | | | | | Motorcycle | | | | | | | | | 1 | 2 |
|  |  |  |  |  |  | | | | | Freeze | | | | | | | | | 1 | 2 |
|  |  |  |  |  |  | | | | | Fan | | | | | | | | | 1 | 2 |
|  |  |  |  |  |  | | | | | Sewing-machine | | | | | | | | | 1 | 2 |
|  |  |  |  |  |  | | | | | OthersSpecify________________________ | | | | | | | | | 1 | 2 |
| 9 | Which type of toilet do you use? | | | | **No.** | | | | | Toilet type | | | | | | | | | **Yes** | **No** |
|  |  |  |  |  | 1 | | | | | Pit Toilet | | | | | | | | | 1 | 2 |
|  |  |  |  |  | 2 | | | | | Paka Toilet | | | | | | | | | 1 | 2 |
|  |  |  |  |  | 3 | | | | | Soil made Toilet | | | | | | | | | 1 | 2 |
|  |  |  |  |  | 4 | | | | | Sanitary | | | | | | | | | 1 | 2 |
|  |  |  |  |  | 5 | | | | | Others Specify]______________________ | | | | | | | | | 1 | 2 |
| 10 | What is the main source of drinking water? | | | | **No.** | | | | | Source type | | | | | | | | |  |  |
|  |  |  |  |  | 1 | | | | | Tube well | | | | | | | | | 1 | 2 |
|  |  |  |  |  | 2 | | | | | Ponds/rivers/puddles/streams | | | | | | | | | 1 | 2 |
|  |  |  |  |  | 3 | | | | | Rain Water | | | | | | | | | 1 | 2 |
|  |  |  |  |  | 4 | | | | | Pipe/tap/Supply Water | | | | | | | | | 1 | 2 |
|  |  |  |  |  | 5 | | | | | Pure/filtered water | | | | | | | | | 1 | 2 |
|  |  |  |  |  | 6 | | | | | Others please specify____________________ | | | | | | | | | 1 | 2 |
| 11 | Do the household have Electricity? | | | | 1. Yes | | | | | 2. No | | | | | | | | |  |  |
| Part 3B: **Other sources of household income** | | | | | | | | | | | | | | | | | | | | |
| 12 | Do the household have any permanent income source? | | | | 1. Yes | | | | | | 2. No | | | | | | | |  |  |
| 14 | Do the household has any child labor? | | | | 1. Yes | | | | | | 2. No | | | | | Not appilicable 99 | | |  |  |
| 15 | Section-2  In the last 12 months, what were the other sources of household irregular income? Exclude the items considered in the section-2 | | | | **No.** | | | | | | Sources of income | | | | | | | | Amount of income in Taka | |
|  |  |  |  |  | 1. | | | | | | Food for work | | | | | | | |  |  |
|  |  |  |  |  | 2. | | | | | | Income from assets | | | | | | | |  |  |
|  |  |  |  |  | 3. | | | | | | VGD/destitute allowance | | | | | | | |  |  |
|  |  |  |  |  | 4. | | | | | | Old allowance | | | | | | | |  |  |
|  |  |  |  |  | 5. | | | | | | Pension | | | | | | | |  |  |
|  |  |  |  |  | 6. | | | | | | Freedom fighter allowance | | | | | | | |  |  |
|  |  |  |  |  | 7. | | | | | | Rremittence | | | | | | | |  |  |
|  |  |  |  |  | 8. | | | | | | Stipend/scholarship | | | | | | | |  |  |
|  |  |  |  |  | 9. | | | | | | Other allowances | | | | | | | |  |  |
|  |  |  |  |  | 10. | | | | | | Others sources specify_____________ | | | | | | | |  |  |
| Part 3C: Loan information of Household | | | | | | | | | | | | | | | | | | | | |
| 16 | In the last 12 months, was any household member involved with loan? | | | | | 1. Yes | | 2. No | | | | | | | | | Code: 2 | | |  |
|  | If yes please fill-up the following section | | | | |  | |  | | | | Where did you invest the borrowed money during the last year?  Code: 2 | | | | | \| 1. Poultry farming \| 10. To buy fishing equipment \| \| --- \| --- \| \| 2. Cow/goat farming \| 11. Buy boat \| \| 3. Handy crafting \| 12. Buy van /rickshaw \| \| 4. Agriculture \| 13. Small business \| \| 5. House building/repairing \| 14. Business \| \| 6. Marriage \| 15. Buy agricultural equipment \| \| 7. To buy consumption goods \| 16. Pay the borrowed amount \| \| 8. For educational expenses \| 17. Sent abroad \| \| 9. For treatment \| 18. Others \| | | | |
|  | **Member code** | | | What is the source of loan?  1. Community scheme  2Small lender organization  3From Bank  4. Other Specify]....... | | How much money did you loan in the last time? | | What is the left-over ammount of your loan? | | | |  |  |  |  |  |  |  |  |  |
|  |  | | |  | |  | |  | | | |  | | | | |  |  |  |  |
|  |  | | |  | |  | |  | | | |  | | | | |  |  |  |  |
|  |  | | |  | |  | |  | | | |  | | | | |  |  |  |  |
|  |  | | |  | |  | |  | | | |  | | | | |  |  |  |  |
| Part 3D:  **Household Food Security related information** | | | | | | | | | | | | | | | | | | | | |
| 18 | How often did you eat three `square meals' full stomach meals a day in the past 12 months not a festival day? | | | | | | 1 Mostly 3 meals each day  2. Sometimes 3 meals per day  3. Rarely 3 meals per day 1-6 times this year  4. Never | | | | | | | | | | | |  |  |
| 19 | In the last 12 months how often did you yourself skip entire meals because there was not enough food? | | | | | | 1. Never  2. Rarely 1-6 times this year  3. Sometimes 7-12 times this year  4. Often few times each month | | | | | | | | | | | |  |  |
| 20 | In the last 12 months how often did you personally eat less food in a meal because there was not enough food? | | | | | | 1. Never  2. Rarely 1-6 times this year  3. Sometimes 7-12 times this year  4. Often few times each month | | | | | | | | | | | |  |  |
| 21 | In the last 12 months, how often did you or any of your family have to eat wheat or another grain although you wanted to eat rice not including when you were sick? | | | | | | 1. Never  2. Rarely 1-6 times this year  3. Sometimes 7-12 times this year  4. Often few times each month | | | | | | | | | | | |  |  |
| 22 | In the past 12 months how often did your family have to ask food from relatives or neighbors to make a meal? | | | | | | 1. Never  2. Rarely 1-6 times this year  3. Sometimes 7-12 times this year  4. Often few times each month | | | | | | | | | | | |  |  |
| 23 | How many months you can live on from your land income? | | | | | | ______Month | | | | | | | | | | | |  |  |
| **Section 4:Household expenditure related information** | | | | | | | | | | | | | | | | | | | | |
| C1 | | C2 | | | | | | | C3 | | | | | | | | | | | |
| Sl. no | | Types of food | | | | | | | Amount consumed If do not consumed put “0” | | | | | | | | | | | |
| **Part A: Food Weekly expenditure** | | | | | | | | | | | | | | | | | | | | |
| **4.1 Rice crop consumed in the last week** | | | | | | | | | | | | | | | | | | | | |
| 1 | | Rice coarse (Kg) | | | | | | |  | | | | | | | | | | | |
| 2 | | Rice-plain (Kg) | | | | | | |  | | | | | | | | | | | |
| 3 | | Wheat (Kg) | | | | | | |  | | | | | | | | | | | |
| 4 | | Flour (Kg) | | | | | | |  | | | | | | | | | | | |
| 5 | | Flattened/Puffed rice (Kg) | | | | | | |  | | | | | | | | | | | |
| **4.2 Pulse crop consumed in the last week** | | | | | | | | | | | | | | | | | | | | |
| 1 | | Lentil (kg) | | | | | | |  | | | | | | | | | | | |
| 2 | | Bengal gram pulses (Kg) | | | | | | |  | | | | | | | | | | | |
| 3 | | Mush Kalai (Kg) | | | | | | |  | | | | | | | | | | | |
| 4 | | Pea gram (Kg) | | | | | | |  | | | | | | | | | | | |
| 5 | | Chick ling-Vetch (Kg) | | | | | | |  | | | | | | | | | | | |
| 6 | | Green gram (Kg) | | | | | | |  | | | | | | | | | | | |
| 7 | | Anchor (Kg) | | | | | | |  | | | | | | | | | | | |
| **4.3 cooking oil consumed in the last week** | | | | | | | | | | | | | | | | | | | | |
| 1 | | Soybean oil (liter) | | | | | | |  | | | | | | | | | | | |
| 2 | | Mustard oil (liter) | | | | | | |  | | | | | | | | | | | |
| 3 | | Dalda (liter) | | | | | | |  | | | | | | | | | | | |
| 4 | | Ghee (Kg) | | | | | | |  | | | | | | | | | | | |
| 5 | | Palm Oil (litter) | | | | | | |  | | | | | | | | | | | |
| 6 | | Others Specify________ | | | | | | |  | | | | | | | | | | | |
| **4.4 leaf vegetables consumed in the last week** | | | | | | | | | | | | | | | | | | | | |
| 1 | | Amaranth (Kg/bundle) | | | | | | |  | | | | | | | | | | | |
| 2 | | Malabar spinach (Kg/bundle) | | | | | | |  | | | | | | | | | | | |
| 3 | | Spinach (Kg/bundle) | | | | | | |  | | | | | | | | | | | |
| 4 | | Spinach ipomoea aquatic (Kg/bundle) | | | | | | |  | | | | | | | | | | | |
| 5 | | Others leafy (Kg/bundle) | | | | | | |  | | | | | | | | | | | |
| 6 | | Others leafy (Kg/bundle) | | | | | | |  | | | | | | | | | | | |
| 7 | | Others leafy (Kg/bundle) | | | | | | |  | | | | | | | | | | | |
| 8 | | Potato (Kg) | | | | | | |  | | | | | | | | | | | |
| 9 | | Pointed gourd (Kg) | | | | | | |  | | | | | | | | | | | |
| 10 | | Bitter melon (Kg) | | | | | | |  | | | | | | | | | | | |
| 11 | | Cauliflower (Kg/piece) | | | | | | |  | | | | | | | | | | | |
| 12 | | Cabbage (Kg/Piece) | | | | | | |  | | | | | | | | | | | |
| 13 | | Brinja (Kg) | | | | | | |  | | | | | | | | | | | |
| 14 | | Arum (Kg) | | | | | | |  | | | | | | | | | | | |
| 15 | | Gourd (Number) | | | | | | |  | | | | | | | | | | | |
| 16 | | Tommato (Kg) | | | | | | |  | | | | | | | | | | | |
| 17 | | Green papaya (Kg) | | | | | | |  | | | | | | | | | | | |
| 18 | | Green Banana (Number) | | | | | | |  | | | | | | | | | | | |
| 19 | | Green Chili (Kg) | | | | | | |  | | | | | | | | | | | |
| 20 | | Onion (Kg) | | | | | | |  | | | | | | | | | | | |
| 21 | | Garlic (kg) | | | | | | |  | | | | | | | | | | | |
| 22 | | Ginger (kg) | | | | | | |  | | | | | | | | | | | |
| 23 | | ______Others vegetables (Kg) | | | | | | |  | | | | | | | | | | | |
| 24 | | _____Others vegetables (Kg) | | | | | | |  | | | | | | | | | | | |
| 25 | | ______Others vegetables (Kg) | | | | | | |  | | | | | | | | | | | |
| **Part B: Food Monthly consumption]** | | | | | | | | | | | | | | | | | | | | |
| **4.5** Spices consumption in the last month | | | | | | | | | | | | | | | | | | | | |
| Sl. no | | | Types of food | | | | | | Amount consumed (If do not consumed put “0”) | | | | | | | | | | | |
| **C1** | | | **C2** | | | | | | **C3** | | | | | | | | | | | |
| 1 | | | Dried chili (gm) | | | | | |  | | | | | | | | | | | |
| 2 | | | Turmeric (gm) | | | | | |  | | | | | | | | | | | |
| 3 | | | Cumin (gm) | | | | | |  | | | | | | | | | | | |
| 4 | | | Cinnamon (gm) | | | | | |  | | | | | | | | | | | |
| 5 | | | Cardamom (gm) | | | | | |  | | | | | | | | | | | |
| 6 | | | Others spice______gm | | | | | |  | | | | | | | | | | | |
| 7 | | | Others spice_______ gm | | | | | |  | | | | | | | | | | | |
| 8 | | | Others spice_______ gm | | | | | |  | | | | | | | | | | | |
| **4.6 Fish, meat, egg and milk consumption in the last month** | | | | | | | | | | | | | | | | | | | | |
| 1 | | | Large Fish (kg) | | | | | |  | | | | | | | | | | | |
| 2 | | | Smaller Fish kg] | | | | | |  | | | | | | | | | | | |
| 3 | | | Dried fish (kg) | | | | | |  | | | | | | | | | | | |
| 4 | | | Beef (kg) | | | | | |  | | | | | | | | | | | |
| 5 | | | Buffalo meat (Kg) | | | | | |  | | | | | | | | | | | |
| 6 | | | Local chicken meat (kg) | | | | | |  | | | | | | | | | | | |
| 7 | | | Poultry chicken meat (Kg) | | | | | |  | | | | | | | | | | | |
| 8 | | | [Duck meat (Kg) | | | | | |  | | | | | | | | | | | |
| 9 | | | Mutton (Kg) | | | | | |  | | | | | | | | | | | |
| 10 | | | Egg (Number) | | | | | |  | | | | | | | | | | | |
| 11 | | | Milk (Litter) | | | | | |  | | | | | | | | | | | |
| 12 | | | Other protein items__ __(Kg) | | | | | |  | | | | | | | | | | | |
| 13 | | | Other protein items__ __(Kg) | | | | | |  | | | | | | | | | | | |
| 14 | | | Other protein items__ __  Kg | | | | | |  | | | | | | | | | | | |
| **4.7 [Fruits consumption in the last months]** | | | | | | | | | | | | | | | | | | | | |
| 1 | | Banana (Number) | | | | | | |  | | | | | | | | | | | |
| 2 | | Chinese (date | | | | | | |  | | | | | | | | | | | |
| 3 | | Olive (Kg) | | | | | | |  | | | | | | | | | | | |
| 4 | | Papyaa (Kg) | | | | | | |  | | | | | | | | | | | |
| 5 | | Mango (Kg) | | | | | | |  | | | | | | | | | | | |
| 6 | | Jackfruit (Number) | | | | | | |  | | | | | | | | | | | |
| 7 | | Java Plum (Kg) | | | | | | |  | | | | | | | | | | | |
| 8 | | Apple (Kg) | | | | | | |  | | | | | | | | | | | |
| 9 | | Orange (Number) | | | | | | |  | | | | | | | | | | | |
| 10 | | Graps (Kg) | | | | | | |  | | | | | | | | | | | |
| 11 | | Guava (Kg) | | | | | | |  | | | | | | | | | | | |
| 12 | | Pineaple (Number) | | | | | | |  | | | | | | | | | | | |
| 13 | | Other fruits _________ Kg | | | | | | |  | | | | | | | | | | | |
| 14 | | Other fruits _________ Kg | | | | | | |  | | | | | | | | | | | |
| 15 | | Other fruits _________ Kg | | | | | | |  | | | | | | | | | | | |
| **4.8 Other foods in the last month** | | | | | | | | | | | | | | | | | | | | |
| 1 | | Sugar (Kg) | | | | | | |  | | | | | | | | | | | |
| 2 | | Jaggery (Kg) | | | | | | |  | | | | | | | | | | | |
| 3 | | MIlk powder (Kg) | | | | | | |  | | | | | | | | | | | |
| 4 | | Salt (Kg) | | | | | | |  | | | | | | | | | | | |
| 5 | | Noodles (Packate) | | | | | | |  | | | | | | | | | | | |
| 6 | | Semolina (Kg) | | | | | | |  | | | | | | | | | | | |
| 7 | | Cold drink (litter) | | | | | | |  | | | | | | | | | | | |
| 8 | | Sweetned food | | | | | | |  | | | | | | | | | | | |
| 9 | | __________________ Child food (Kg) | | | | | | |  | | | | | | | | | | | |
| 10 | | Tea leaf (Kg) | | | | | | |  | | | | | | | | | | | |
| 11 | | Biscut (Pack) | | | | | | |  | | | | | | | | | | | |
| 12 | | Bombay mix (Packet) | | | | | | |  | | | | | | | | | | | |
| 13 | | Others_________ Number | | | | | | |  | | | | | | | | | | | |
| 14 | | Others_________ Number | | | | | | |  | | | | | | | | | | | |
| 15 | | Others_________ Number | | | | | | |  | | | | | | | | | | | |
| **Part C:** Tobacco products Weekly expense | | | | | | | | | | | | | | | | | | | | |
| **4.9** Tobacco intact in last week | | | | | | | | | | | | | | | | | | | | |
| 1 | | Batel leaf (number) | | | | | | |  | | | | | | | | | | | |
| 2 | | Betel nut (number) | | | | | | |  | | | | | | | | | | | |
| 3 | | Tobbacco leaf (Kouta) | | | | | | |  | | | | | | | | | | | |
| 4 | | Tobbacco (number) | | | | | | |  | | | | | | | | | | | |
| 5 | | Gul (Kouta) | | | | | | |  | | | | | | | | | | | |
| 6 | | Biri (Packet) | | | | | | |  | | | | | | | | | | | |
| 7 | | Cigaret (Packet) | | | | | | |  | | | | | | | | | | | |
| 8 | | Others_________________ Number | | | | | | |  | | | | | | | | | | | |
| 9 | | Others_________________ Number | | | | | | |  | | | | | | | | | | | |
| 10 | | Others_________________ Number | | | | | | |  | | | | | | | | | | | |

| **Part D: [Non-food Monthly expenditure]** | | |
| --- | --- | --- |
| **C**1 | **C2** | **C**3 |
| **Sl. no** | **Types** | **Expenditure ammount Monthly** |
| **4.10 Non food expenditure in last month** | | |
| 1 | House rent |  |
| 2 | Electricity Bill/Solar Power |  |
| 3 | Gas bill |  |
| 4 | Fuel wood |  |
| 5 | Fuel oil (kerosene, petrol) |  |
| 6 | **Transport** |  |
| 7 | Health |  |
| 8 | Education school fees, (coaching, private teacher, etc.) |  |
| 9 | Notebooks/Pens/books/other study materials] |  |
| 10 | Communication expense (mobile, telephone, etc.) |  |
| 11 | Dish bills/internet bill |  |
| 12 | Cosmetics expense (Cream, Powder, make-up etc.) |  |
| 13 | Hair Oil/Coconut Oil |  |
| 14 | Cleaning and hygiene (soap, detergent, shampoo etc.) |  |
| 15 | Laundry |  |
| 16 | Co-operative instalment/ Micro-credit instalment |  |
| 17 | Salon/Spa |  |
| 18 | Others Specify |  |

| **Part E: [Non-food Yearly expenditure]** | | |  |
| --- | --- | --- | --- |
| **4.11 Non-food expenditure in last year** | | |  |
| **Sl.no** | **Types** | **Expenditure amount in the last year** |  |
| 1 | Clothing |  |  |
| 2 | Shoe |  |  |
| 3 | Quilt/quilt/pillow/blanket/bed |  |  |
| 4 | Entertainment/religious festival |  |  |
| 5 | House construction/House Renovation |  |  |
| 6 | Furniture |  |  |
| 7 | Insurance Premium |  |  |
| 8 | House keeper/ maids salary |  |  |
| 9 | **Gifts provide** |  |  |
| 10 | Donation |  |  |
| 11 | Travel |  |  |
| 12 | Others Specify |  |  |

|  | **Section 5: Treatment and expenditure related information of the household members in the last three months (Write individual information in separate row)** | | | | | | | | | | | | | | | | | | | | | | | | | | | | | |
| --- | --- | --- | --- | --- | --- | --- | --- | --- | --- | --- | --- | --- | --- | --- | --- | --- | --- | --- | --- | --- | --- | --- | --- | --- | --- | --- | --- | --- | --- | --- |
|  | **Does any household member got sick or have symptoms of sickness in the last three months? 1. Yes 2. No** | | | | | | | | | | | | | | | | | | | | | | | | | | | | | |
| 1 | | 2 | 3 | 4 | 5 | 6 | | 7 |  | 8 | 9 | | | | | | | | | | 10 | 11 | 12 | 13 | 14 | | | | | |
| Member code | | The disease code of the member | How many days you/ the member suffered from illness? | Did you or the member sought treatment for that disease?  Code: Yes1 No2 If answer is no Only question 5 is applicable | If you didn’t seek treatment, why?   \| 1. Problem was not severe \| \| --- \| \| 2. The cost for treatment was very high \| \| 3. Didn’t have enough money \| \| 4. Long distance of the health facility \| \| 5. No one was available to take to the facility \| \| 6. Didn’t know the location of facility \| \| 7. [If others, specify] \| \|  \| \|  \| | \| Where did you seek treatment from? \| \| --- \| \| 1.  Medical College Hospital \| \| 2. Specialized hospital \| \| 3. [Referral district hospital] \| \| 4. Other District Hospital \| \| 5. Upazila Health Complex \| \| 6.Union Sub center/CC/Rural dispensary \| \| 7. Maternal and Child Welfare Center \| \| 8. Private Clinic and Hospital \| \| 9. NGO Clinic/ Hospital \| \| 10. NGO Health Worker \| \| 11. Government Health Worker \| \| 12. Qualified Private Practitionar \| \| 13. Unqalified Private Practitionar \| \| 14. Ayurvedic /homeopathic \| \| 15. Self-treatment \| \| 16. Pharmacy \| \| 17. OthersSpecify...] \| | | [Did you get admitted to the hospital for that problem? Code: Yes1 No 2; If no then go the question no 9] | If yes, how many days you were admitted to the hospital? in full days] | [Was your treatment covered by the SSK?] | [Cost for treatment If any item is not applicable, palce “0”] | | | | | | | | | | [How many days did you absent from your work due to illness? Full days] | [In the last three months, despite of illness how many days were you present at your work? write “99” if not applicable] | [In the last three months, did you left any work permanently due to illness? yes1, No 2, Not appicable 99] | In the last 12 months, did any of the household member had to leave school permanently? Yes 1 No 2 Not applicable 3 | How did you manage the treatment cost?   \| 1. Regular income \| \| --- \| \| 2. Family savings \| \| 3. SSK provided \| \| 4. Insurance \| \| 5. Selling of own materials \| \| 6. Selling of Trees/Agricultural crops/domestic animals \| \| 7. Selling of permanent property \| \| 8. Mortgage of land or property \| \| 9. Borrowing money from lender \| \| 10. Help from friends or relatives \| \| 11. Borrow from Relatives/friends/colleagues \| \| 12. Loan from institute/association \| \| 13. Others, Please specify \| | | | | | |
|  |  |  |  |  |  |  |  |  |  |  | Consultaiton fee | Medicine | Bed rent | Diagnostic | Transport | Tips | Attednant | Package | Operation | Others |  |  |  |  |  | | | | | |
|  |  |  |  |  |  |  |  |  |  |  |  |  |  |  |  |  |  |  |  |  |  |  |  |  | উৎস-1 | টা | উৎস-2 | টা | উৎস-3 | টা |
|  |  |  |  |  |  | Sl. no | Source of treatment |  |  |  |  |  |  |  |  |  |  |  |  |  |  |  |  |  |  |  |  |  |  |  |
|  | |  |  |  |  | 1. |  |  |  |  |  |  |  |  |  |  |  |  |  |  |  |  |  |  |  |  |  |  |  |  |
|  |  |  |  |  |  | **2.** |  |  |  |  |  |  |  |  |  |  |  |  |  |  |  |  |  |  |  |  |  |  |  |  |
|  |  |  |  |  |  | **3.** |  |  |  |  |  |  |  |  |  |  |  |  |  |  |  |  |  |  |  |  |  |  |  |  |
|  | |  |  |  |  | **1.** |  |  |  |  |  |  |  |  |  |  |  |  |  |  |  |  |  |  |  |  |  |  |  |  |
|  |  |  |  |  |  | **2.** |  |  |  |  |  |  |  |  |  |  |  |  |  |  |  |  |  |  |  |  |  |  |  |  |
|  |  |  |  |  |  | **3.** |  |  |  |  |  |  |  |  |  |  |  |  |  |  |  |  |  |  |  |  |  |  |  |  |
|  |  |  |  |  |  | **2.** |  |  |  |  |  |  |  |  |  |  |  |  |  |  |  |  |  |  |  |  |  |  |  |  |
|  |  |  |  |  |  | **3.** |  |  |  |  |  |  |  |  |  |  |  |  |  |  |  |  |  |  |  |  |  |  |  |  |
|  | * 1. Cough 2. Fever 3. Malaria 4. Typhoid 5. Diarrhoea/dysentery 6. Vomiting 7. Asthma/breathlessness 8. Minor injury 9. Bone fracture 10. Jaundice 11. Weakness 12. Worms 13. Pain/discomfort 14. Skin disease 15. Convulsion/Seizure 16. Dyspepsia 17. Hypertension 18. Diabetes 19. Tuberculosis 20. Leprosy  21. Injury/Paralysis 22. Arthritis 23. Rheumatic Fever 24. Cancer 25. Delivery 26. Obstetric emergency 27. Mental problem 28. Others | | | | | | | | | | | | | | | | | | | | | | | | | | | | | |

| **Section6: Effect of illness on the food security**  If no member of the household suffers from any illness, start from the next section | | |
| --- | --- | --- |
| **Sl.no** | **Question** | **Type of Answer** |
| 1 | In the last three months, did your family have to squeeze consumption expenditure due to the illness of any member? | 1. Yes  2. No  99. Not applicable |
| 2 | In the last three months, did your family have to squeeze other expenditure e.g., education, clothing, furniture due to the illness of any member? | 1. Yes  2. No  99. Not applicable |

**Section 7: Effect of Health expenditure and SSK related Question**

| **Sl.no1** | **Question 2** | | **Type of answer 3** |
| --- | --- | --- | --- |
| 1 | Do you think that many people faces financial catastrophe while seeking healthcare service? | | 1. Yes 2. No |
| 2 | Do you think, some of your neighbour used to work despite of their illness? | | 1. Yes 2. No |
| 3 | Do you know, different institutions used to bear treatment cost of its members? | | 1. Yes 2. No |
| ~~4~~ | Have you ever heard of life insurance? | | 1. Yes 2. No |
| 5 | Have you ever heard of health insurance? | | 1. Yes 2. No |
| 6 | Do you know, there is a health protection scheme in this area for the poor people? | | 1. Yes 2. No |
| 7 | Do you know, how SSK services are provided from the healthcare facility? | | 1. By card  2. By Cash  3. Don’t know |
| 8 | Do you know, how many disease are covered under SSK scheme? | | 1. Yes  2._____ No (If yes, ask how many?) |
| 9 | What types of treatment expenses are borne by the SSK? Multiple responses are acceptable | | 1. Free medicine  2. Free diagnostic/test  3. Referral facility  4. Provide expense for referral to District hospital  5. Free outpatient registration |
| 10 | Where did you learn about the SSK? | | 1. From others in your locality  2. Representative from SSK  3. From familiar person  4. From local leader  5. From Miking  6. From poster  7. From billboard  8. From Television commercial  9. Others, please specify |
| **Part 7A:** SSK service related information | | | |
| 11 | Card number of SSKFill from the card | \|___\|___\|\|___\|___\|\|___\|___\|\|___\|___\|\|___\|___\|\|___\|___\|\|___\|___\|\|___\|___\|___\| | |
| 12 | Card accepting date | \|___\|___\|:\|___\|___\|:\|___\|___\|___\|___\| Day/Month/Year | |
| 13 | Write the code of card issued person from **Section 2** | \|___\|___\| Member code | |
| 14 | Did you ever use SSK card? if didn’t why? | 1. Yes | 2. No (go to the next section) |
| 14.1 | If No, why did you not use the card? Multiple responses are acceptable | | 1. Don’t know how to use  2. Long distance of the facility  3. Poor transportation system  4. Didn’t have need  5. Other |
| 15 | If Yes, How many times did you use the card? | __________ times | |

| **Section 8: Recent Health related question**  In the below table, Each group has three boxes, put tick marks only in one of the boxes that indicate your physical condition. | | | | | | | | | | | | |
| --- | --- | --- | --- | --- | --- | --- | --- | --- | --- | --- | --- | --- |
| Code: | | |  |  |  |  |  |  |  |  |  |  |
| 1 | **walking**  1) I have no problem in walking  3) I have problem in walking  3) Cannot move from bed | | | | | | | | | | | |
| 2 | **Self-care**  1) I have no problem with self-care  2) I have problems washing or dressing myself  3) I am unable to wash or dress myself | | | | | | | | | | | |
| 3 | **Usual activities**  1) I have no problem with performing my usual activities  2) I have some problems with performing my usual activities  3) I am unable to perform my usual activities | | | | | | | | | | | |
| 4 | **Pain/ Discomfort**  1) I have no pain or discomfort  2) I have moderate pain or discomfort  3) I am extreme pain or discomfort | | | | | | | | | | | |
| 5 | **Anxiety/ Depression**  1) I am not anxious or depressed  2) I am moderately anxious or depressed  3) I am extremely anxious or depressed | | | | | | | | | | | |
| 6 | | To help people say how good or bad a health state is, we have drawn a scale (rather like a thermometer) on which the best state you can imagine is marked 100 and the worst state you can imagine is marked 0. We would like to indicate on this scale how good or bad your own health is today, in your opinion. Please do this by drawing a line from the box below to whichever point on the scale indicates how good or bad your health state is today.  Your own health state today | | | | | | | | | | |
| **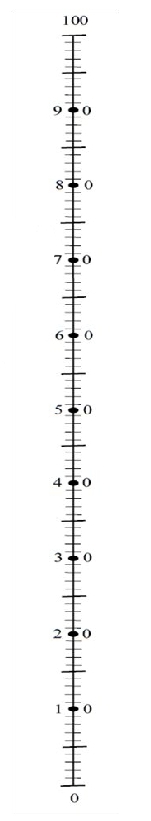**  **Indicate todays physical condition in this scale** | |  |  |  |  |  |  |  |  |  |  |  |

Thank You
